# Breaking paracyclophane: the unexpected formation of non-symmetric disubstituted nitro[2.2]metaparacyclophanes

**DOI:** 10.3762/bjoc.17.109

**Published:** 2021-06-29

**Authors:** Suraj Patel, Tyson N Dais, Paul G Plieger, Gareth J Rowlands

**Affiliations:** 1 School of Fundamental Sciences, Massey University, Private Bag 11 222, Palmerston North 4442, New Zealand

**Keywords:** cyclophane, metaparacyclophane, nitration, paracyclophane, rearrangement

## Abstract

Substituted [2.2]metaparacyclophanes are amongst the least studied of the simple cyclophanes. This is undoubtedly the result of the lengthy syntheses of these compounds. We report the simple synthesis of a rare example of a non-symmetric [2.2]metaparacyclophane. Treatment of [2.2]paracyclophane under standard nitration conditions gives a mixture of 4-nitro[2.2]paracyclophane, 4-hydroxy-5-nitro[2.2]metaparacyclophane and a cyclohexadienone cyclophane.

## Introduction

Cyclophanes have been described as having bent and battered benzene rings [1] due to a structure that involves one, or more, aromatic rings linked by aliphatic chains at non-adjacent carbon positions. Their constrained three-dimensional shapes enforce unusual conformations and interactions between the aromatic decks, all of which results in their unique properties [2–5].

**Figure 1:**
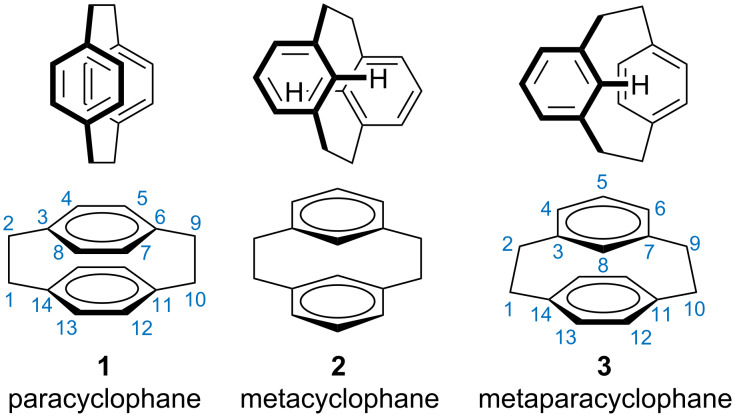
The common [2.2]cyclophanes.

The most studied cyclophane is [2.2]paracyclophane (**1**, Figure 1). Not only is it the archetypal cyclophane, with a strong interaction between the two aromatic rings, but it is readily available, being a ‘dimer’ for the polymer parylene [6–7]. Over the last twenty years, there has been a resurgence in interest in this compound as a scaffold for the synthesis of asymmetric catalysts, energy materials, and as the basis of the study of through-space conjugation [2,8–16]. There are fewer studies of [2.2]metacyclophane (**2**) and its derivatives. This is probably related to a lack availability as well as the reduced interaction between the aromatic rings [17].

But the odd one out of the simple [2.2]cyclophane series appears to be [2.2]metaparacyclophane (**3**). Compared to the other two isomers, there has been scant research [18–23] conducted on this framework. A search for the word `[2.2]metacyclophane*` in SciFinder gives 315 hits (192 hits on Web of Science) while a search for word `[2.2]metaparacyclophane*` only has 69 hits on Scifinder (and 35 for Web of Science; searches were conducted 29th September 2020). Only a single group regularly publishes in this area [24–31]. Yet [2.2]metaparacyclophane (**3**) has a fascinating structure that mix the characteristics of both meta- and paracyclophane [32]. The carbon at the 8-position of the *meta* ring is forced over the *para* ring, leading to distinct chemical shifts for substituents. Its strain energy (23 kcal mol^−1^) places it between [2.2]paracyclophane (**1**) and [2.2]metacyclophane (**2**) (31 kcal mol^−1^ and 13 kcal mol^−1^, respectively) [33–34].

[2.2]Metaparacyclophane (**3**) was first isolated by Cram et al., the pioneer of [2.2]cyclophane chemistry, by the acid-catalyzed rearrangement of [2.2]paracyclophane (**1**) [35–36]. This methodology furnished the non-substituted cyclophane. A more general route to [2.2]metaparacyclophane (**3**) derivatives involves synthesis of 2,11-dithia[3.3]metaparacyclophane followed by the extrusion of sulfur, or more commonly sulfur dioxide, and this has become the de facto route to these molecules [25,37–43]. Even though this chemistry can produce any substitution pattern there are few unsymmetrical [2.2]metaparacyclophanes (**3**) and only one X-ray crystallographic structure found in the CCDC database, and this is a triple-layered cyclophane [44].

We have been interested in the formation of substituted [2.2]paracyclophanes for a number of years [45–52], and have recently focused on 4-amino[2.2]paracyclophanes [14,53–55]. For the most part, we have avoided nitration. The literature is full of many different procedures, and the results can be unpredictable [55–60]. Yet nitration obviously presents one of the most direct routes to 4-amino[2.2]paracyclophanes so we returned to this venerable reaction. In this paper, we disclose a simple synthesis of 4-hydroxy-5-nitro[2.2]metaparacyclophane (**5**), a side-product from our nitration reactions. This chemistry offers a rapid route to non-symmetric functionalized [2.2]metaparacyclophanes.

## Results and Discussion

**Scheme 1:**
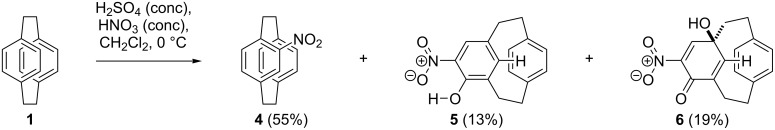
Nitration of [2.2]paracyclophane (**1**) and the synthesis of 4-hydroxy-5-nitro[2.2]metaparacyclophane (**5**) and the cyclohexadienone cyclophane **6** (average yield from more than three repeats quoted).

The nitration of [2.2]paracyclophane (**1**) is rarely a clean reaction [55–60], and the side-products are believed to include overnitration, as a mixture of regioisomers, as well as the products of oxidation and polymerization. Extensive optimization led us to a set of conditions that routinely provided 4-nitro[2.2]paracyclophane (**4**) in good yields regardless of the scale of reaction (nitration has been performed on scales ranging from 0.5 g to 30 g). The ^1^H NMR of the crude reaction mixture invariably shows two other products that displayed surprisingly high field, yet distinct ^1^H NMR signals at 5.80 and 5.62 ppm, along with the desired nitro[2.2]paracyclophane (**4**). The ratio of products varies with concentration. When the nitration was conducted on a large scale and at relatively high concentrations the ratio of nitro[2.2]paracyclophane (**4**) to the side-products was in the range of 4:1:1 (Scheme 1).

**Figure 2:**
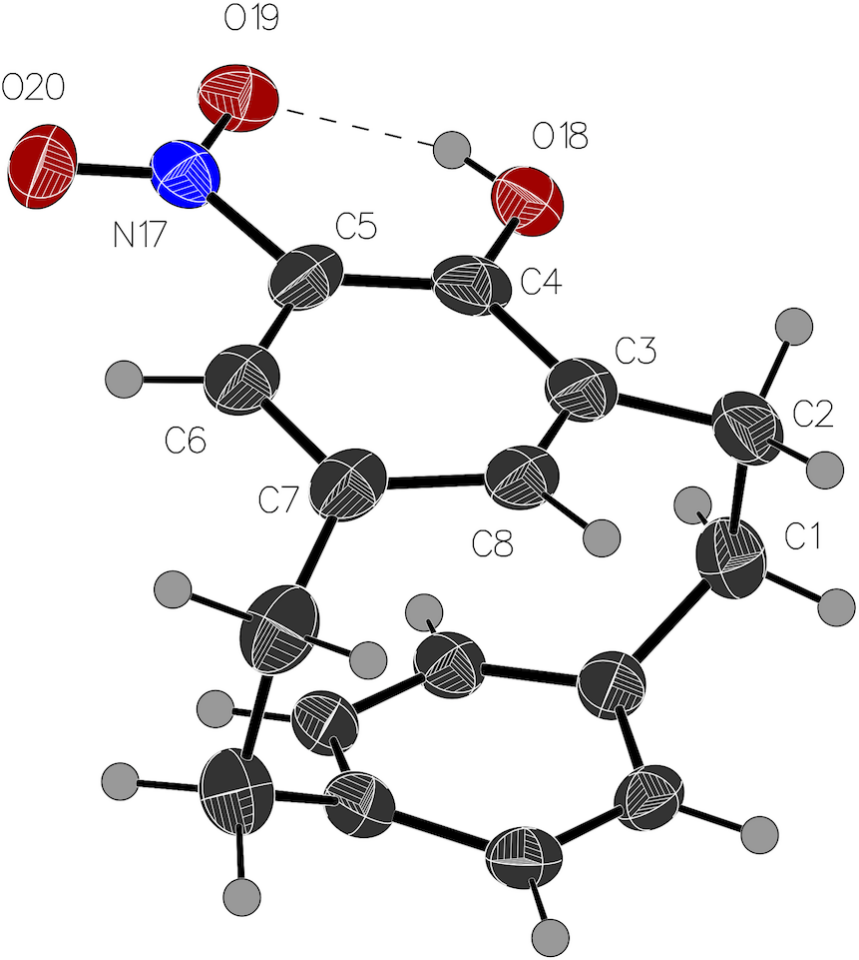
Crystal structure of **5**. Ellipsoids are drawn at a 50% probability level [63–66].

**Figure 3:**
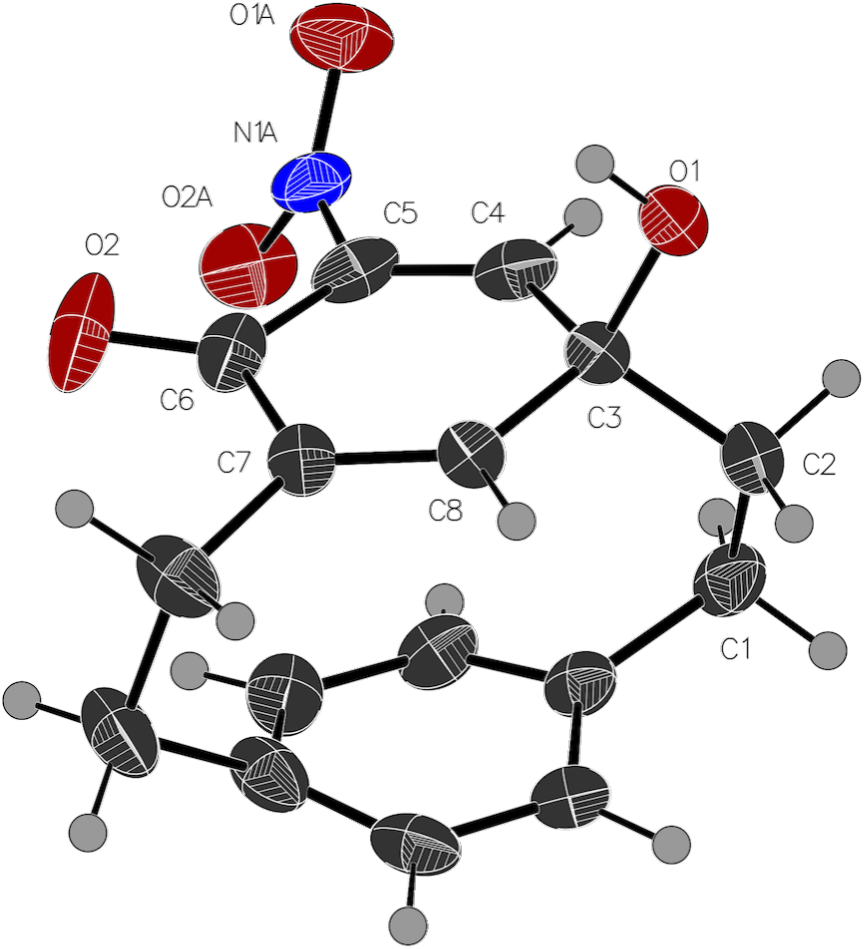
Crystal structure of **6**. Ellipsoids are drawn at a 50% probability level [63].

One, **5**, was clearly an isomer of a hydroxynitro[2.2]paracyclophane with characteristic ^1^H NMR signal at 10.79 ppm for an internal hydrogen bond, and a signal at 7.53 ppm for C–H *ortho* to the nitro group. But the upfield signal at 5.80 ppm, along with the unusual splitting of the bridgehead protons (H1, 2, 9 and 10) was unlike any [2.2]paracyclophane derivative we had observed. It was clear that we had isolated a cyclophane, but we suspected rearrangement [61–62]. The structure of the other side-product, **6**, was even harder to determine due to the lack of characteristic high field aromatic proton signals. Fortunately, both molecules are crystalline solids, and X-ray crystallographic analysis revealed that one molecule is 4-hydroxy-5-nitro[2.2]metaparacyclophane (**5**, Figure 2), while the other was a cyclohexadienone cyclophane (**6**, Figure 3). The former is, to the best of our knowledge, the first example of a crystal structure of a non-symmetric [2.2]metaparacyclophane.

The structure of the disubstituted [2.2]metaparacyclophane (**5**) is analogous to that of the unsubstituted [2.2]metaparacyclophane (**3**). The angle between the two aromatic rings as defined by carbon atoms 3–7 on the *meta*-ring and 12, 13, 15, 16 on the *para*-ring is 13.1° for [2.2]metaparacyclophane (**3**) [32], and 13.9° for the disubstituted derivative **5**. The nitro group is held in the plane of the *meta*-ring by hydrogen bonding with the hydroxy group at C4.

In the cyclohexadienone cyclophane **6**, with its extra sp^3^-hybridized atom to the bridge, the distortion is reduced, the angle between the planes is just 7°. The conformation of the nitro group has changed. In the absence of a hydroxy group for hydrogen bonding, there is a repulsion between the nitro oxygen atom and the lone pair of electrons on the dienone. The nitro group is now 60.4° to the cyclohexadienone ring. It is not unusual for a nitro group to be rotated out of coplanarity with a substituted aromatic ring [24,37].

We were curious to know how these compounds formed. It seemed unlikely that rearrangement to [2.2]metaparacyclophane (**3**) occurred prior to nitration. Firstly, no [2.2]metaparacyclophane (**3**) was ever observed in our nitration reaction mixtures. Secondly, Cram has reported that the major product of both bromination and acylation of [2.2]metaparacyclophane (**3**) arises from electrophilic addition to the *para*-substituted ring [36]. Substitution at the 4-position of the *meta*-ring is only the minor product. We attempted to confirm this hypothesis by preparing [2.2]metaparacyclophane (**3**) by the triflic acid-mediated rearrangement of [2.2]paracyclophane (**1**) but were unable to isolate a pure sample. Our best conversion, judged by ^1^H NMR, gave 21% of **3** along with unreacted **1**. The two unsubstituted isomeric cyclophanes, **1** and **3**, could not be separated by standard chromatographic techniques.

Next, we attempted the rearrangement of a range of 4-substituted [2.2]paracyclophanes, including 4-hydroxy-, 4-nitro-, and 4-bromo[2.2]paracyclophane, with either triflic acid or under our standard nitration conditions. The distinct singlet for the C8 proton of **5**, and the slightly higher shift for the bridgehead methylene protons, was not observed in the ^1^H NMR spectra of any of the reactions suggesting rearrangement does not occur. Reaction of the electron-rich 4-hydroxy[2.2]paracyclophane led to decomposition while the electron-poor derivatives barely reacted.

Treatment of [2.2]paracyclophane (**1**) with various acids (nitric, sulfuric, perchloric, and acetic acid) led to differing results. Reaction of **1** with nitric acid alone led to a surprisingly clean, nitration, albeit by a very slow reaction. As stated earlier, nitration is normally a messy reaction. Presumably, the low concentration of nitronium ion present in equilibrium with the acid promotes a clean reaction without polymerization or oxidation. Treatment with sulfuric acid returned unreacted starting material along with a trace of what is almost certainly, according to mass spectrometry, a bis(sulfonic acid), although with the quantities isolated, we were unable to determine which isomer.

Reaction with triflic acid or perchloric acid showed traces of [2.2]metaparacyclophane (**3**). A quick investigation of the strengths of acids required to cause rearrangement revealed that both triflic acid (p*K*_a_ = −14) and perchloric acid (p*K*_a_ = −10) were sufficiently strong to cause rearrangement while nitric (−1.3), sulfuric (−3.0), and acetic acids (4.8) were all too weak. While these results seem to make sense, when we compare the p*K*_a_ values, they do not explain why a mixture of nitric and sulfuric acids causes rearrangement. It is possible that we are comparing the wrong values. p*K*_a_ measures acidity in water. Under our reaction conditions it might better to compare the Hammett acidity function, *H*_0_, as this is more suitable for concentrated acids. On this scale triflic acid *H*_0_ = −14.1, perchloric acid *H*_0_ = −13, and sulfuric acid *H*_0_ = −12.0 are more similar and might explain why nitration conditions cause rearrangement [67]. Alternatively, protonation might not be the key step, and the highly oxidizing nature of nitration conditions that can lead to the formation of a cationic intermediate via a radical cation might control this reaction [68].

**Scheme 2:**
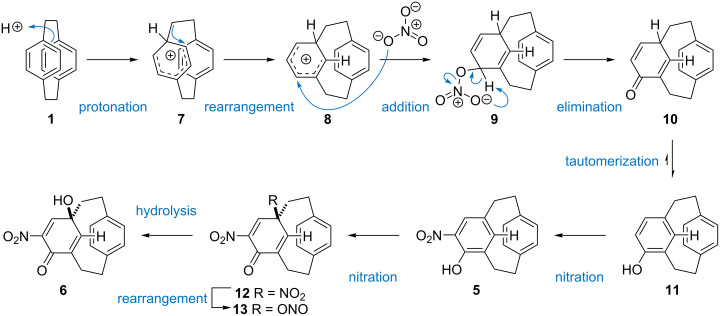
Possible mechanism for the formation of [2.2]metaparacyclophane **5** and cyclohexadienone cyclophane **6** from [2.2]paracyclophane **1**.

A possible mechanism for the formation of **5** and **6** starts with protonation of **1** give the Wheland intermediate or arenium ion **7** (Scheme 2). This occurs at the bridge as this offers greatest release of strain energy. Rearrangement to **8**, as described by Cram and Hefelfinger, further lowers the strain energy of the cyclophane [36]. The carbocation could be trapped by a nitrate anion to give intermediate **9**, which would collapse to the dienone **10**. Tautomerization results in regeneration of the aromatic ring in **11**. Such a route might explain the unique ability of nitration conditions to deliver the substituted [2.2]metaparacyclophane. Alternatively, carbocation **8** might be trapped with water and, under the highly oxidizing conditions, dehydrogenation of the resulting cyclohexadienol would give **11**.The electron-rich 4-hydroxy[2.2]metaparacyclophane (**9**) participates in *ortho* selective nitration to give **5**.

Nitric acid has previously been used to oxidize phenols to cyclohexadienones [69–70], and a plausible pathway involves electrophilic addition *para* to the phenol to form the *ipso*-substituted nitro **12** compound. Subsequent rearrangement of the nitro species **12** to the nitrito dienone **13**, by homolysis and recombination of the radical pair, is followed by hydrolysis to furnish alcohol **6** [70]. Addition of the nitronium ion must occur *anti* to the *para* ring of the cyclophane. Approach from the opposite face is blocked by the lower deck. Once oxidation has occurred conformational flipping of the *meta* deck is impossible and the planar chirality is locked [39].

**Scheme 3:**
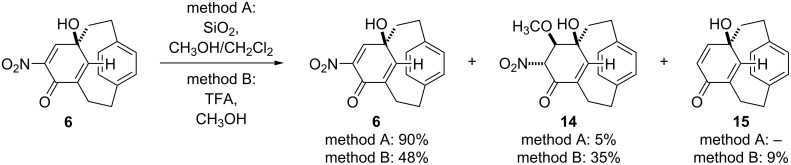
Conjugate addition of methanol and subsequent elimination.

**Figure 4:**
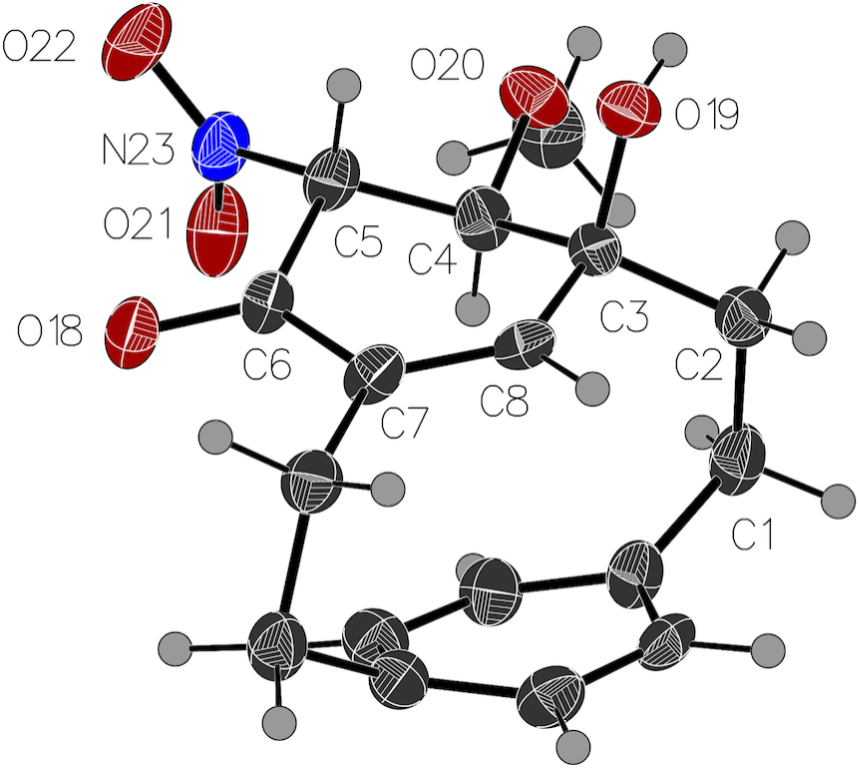
Crystal structure of **14**. Ellipsoids are drawn at a 50% probability level [63].

**Figure 5:**
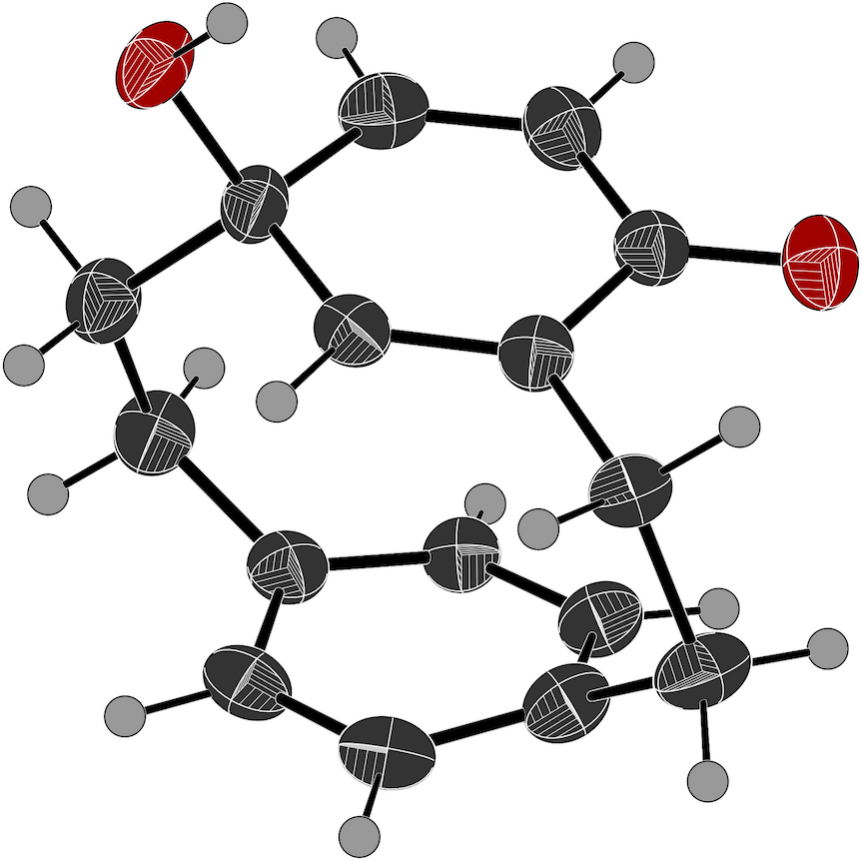
Crystal structure of **15**. Ellipsoids are drawn at a 50% probability level [63].

It is possible that more cyclohexadienone cyclophane **6** is formed than we isolate. The compound can react further. Simply treating it with silica and methanol during filtration leads to the conjugate addition of methanol to the doubly activated alkene to give a cyclohexenone cyclophane **14** (Scheme 3). Attempting to force this reaction with acid leads to a mixture of starting material **6**, the conjugate addition product **14**, and a trace of the denitration product **15**. The structure of both compounds was confirmed by single X-ray crystallography (Figure 4 and Figure 5).

**Figure 6:**
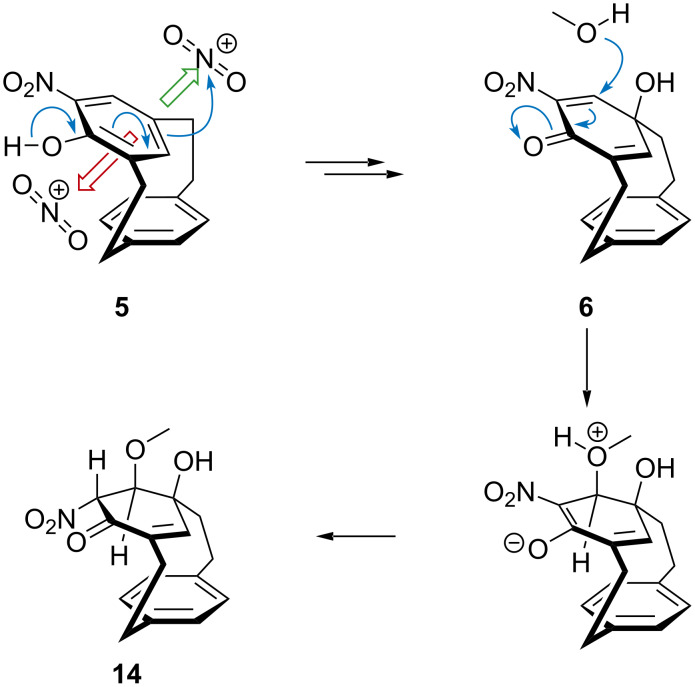
Possible origin of stereoselectivity.

The addition of methanol is stereoselective with only a single diastereomer of **14** being observed (Figure 6). As with the oxidation, we assume that the lower deck blocks approach from one face. Protonation of the adduct also occurs *anti* to the *para* ring. We suspect that this second addition favors the *trans* product over the *cis* rather than the *para* ring influencing the approach of the proton source.

Compound **14** could only be purified by recrystallization; all attempts to purify **14** by column chromatography led to complex mixtures of which the only identifiable product was **15**. Even recrystallization can be problematic. Heating **14** leads to an elimination reaction that re-forms the initial compound **6**. It is unclear how the denitration product **15** forms. Elimination of the ether **14** to regenerate the enone **6** is straightforward, but reductive denitration is a taxing reaction that normally requires strongly reducing conditions or a single electron donor. There are examples of milder denitrations but these are substrate specific [71–72].

## Conclusion

Currently, substituted [2.2]metaparacyclophanes are synthesized through a long sequence involving either high temperature extrusion of sulfur dioxide or photoextrusion of sulfur. We have found a serendipitous route to a disubstituted [2.2]metaparacyclophane. The yield is not high, but the reaction can be performed on a large scale using readily available [2.2]paracyclophane (**1**). This reaction permits rapid access to substituted [2.2]metaparacyclophanes, opening the way for more in-depth study of this fascinating family of compounds.

## Experimental

Solvents and reagents were received from commercial sources (Merck/Sigma-Aldrich, ThermoFisher) without additional purification. [2.2]Paracyclophane was purchased from Curtiss-Wright Surface Technologies. All reactions were performed in oven-dried glassware under atmospheric conditions. Column chromatography was carried out on silica gel (grade 60, mesh size 230–400, Scharlau). Visualization techniques for TLC plates include using ultraviolet light (254 nm) and KMnO_4_. NMR spectra were collected at room temperature on Bruker-500, or Bruker-700 spectrometers and calibrated to the appropriate solvent. ESIMS was recorded on a Dionex UltiMate 3000. High-resolution mass spectrometry was performed using a ThermoScientific Q Exactive Focus Hybrid Quadrupole-Orbitrap mass spectrometer or a Bruker Daltonics MicrOTOF spectrometer. Infrared spectroscopy of compounds was completed on a ThermoFisher Nicolet iS5 with an iD7 ATR accessory. Melting points were analysed on a Gallenkamp melting point instrument.

**4-Nitro[2.2]paracyclophane (4), 5-nitro-4-hydroxy[2.2]metaparacyclophane (5) and (4(16)*Z*)-8-hydroxy-6-nitrotricyclo[9.2.2.14,8]hexadeca-1(13),4,(16),6,11,14-pentaen-5-one (6):** To a solution of [2.2]paracyclophane **1** (10.00 g, 48.06 mmol, 1.0 equiv) in CH_2_Cl_2_ (0.20 M, 240 mL) at 0 °C was added a solution of HNO_3_ (4.00 mL, 96.2 mmol, 2.0 equiv) and H_2_SO_4_ (10.10 mL, 192.3 mmol, 4.0 equiv). The reaction mixture was stirred at 0 °C for 8 h, observing a colour change from clear to yellow. The reaction was poured onto ice (100 g) through filter paper, and stirred until the ice had melted. The layers were separated and the aqueous layer was extracted with CH_2_Cl_2_ (5 × 80 mL). The combined organic layers were dried (MgSO_4_) and concentrated under reduced pressure to yield an orange solid, which was purified by silica-gel chromatography (100% hexane), to furnish (±)-**4** as a yellow solid (6.70 g, 26.5 mmol, 55%), (±)-**5** as a yellow crystalline solid (1.68 g, 6.24 mmol, 13%), and **6** as light yellow crystals (2.61 g, 9.15 mmol, 19%).

**4-Nitro[2.2]paracyclophane (4) **^1^H NMR (500 MHz, CDCl_3_) δ (ppm) 7.22 (d, *J* = 1.2 Hz, 1H, H-5), 6.79 (dd, *J* = 1.3, 7.8 Hz, 1H, H-13), 6.63–6.61 (m, 2H, H-7, H-16), 6.58 (dd, *J* = 1.2, 7.8 Hz, 1H, H-8), 6.53 (dd, *J* = 1.2, 7.7 Hz, 1H, H-15), 6.48 (d, *J* = 6.90 Hz, 1H, H-12), 4.02 (ddd, *J* =1.3, 9.6, 13.4 Hz, 1H, H-2_b_), 3.21–3.13 (m, 4H, H-9, H-10), 3.10–3.03 (m, 2H, H-1_a_, H-2_a_), 2.90 (ddd, *J* = 7.2, 8.5, 13.3 Hz, 1H, H-1_b_); ^13^C NMR (126 MHz, CDCl_3_) δ (ppm) 149.0, 142.2, 139.9, 139.5, 137.9, 137.5, 136.6, 133.3, 133.3, 132.6, 130.1, 129.7, 36.2, 35.1, 35.0, 34.6; IR: 3009, 2928, 1694, 1531, 1516, 1336, 808 cm^−1^; mp: 158–160 °C; *R*_f_: 0.43 (10% EtOAc, 90% hexane). Data matches previous reports [14].

**4-Hydroxy-5-nitro[2.2]metaparacyclophane (5) **^1^H NMR (500 MHz, CDCl_3_) δ (ppm) 10.79 (s, 1H, OH), 7.53 (s, 1H, H-6), 7.24 (d, *J* = 7.8 Hz, 2H, H-13), 7.20 (d, *J* = 7.9 Hz, 1H, H-12), 6.12 (d, *J* = 7.9 Hz, 1H, H-15), 5.98 (d, *J* = 7.9 Hz, 1H, H-16), 5.80 (s, 1H, H-8), 3.23–3.12 (m, 3H, 3 × CH), 2.85–2.76 (m, 2H, 2 × CH), 2.52–2.46 (m, 1H, CH), 2.19–2.12 (m, 1H, CH), 1.99–1.93 (m, 1H, CH); ^13^C NMR (126 MHz, CDCl_3_) δ (ppm) 152.6, 141.2, 140.3, 138.6, 132.5, 132.3, 131.3, 130.8, 130.0, 128.9, 128.1, 121.2, 37.1, 35.8, 35.0, 31.9; HRMS-EI *m/z*: [M]^–^ calcd for C_16_H_14_NO_3_, 268.0968; found, 268.0981; ESIMS (*m/z*): [M]^–^ 264, 252, 223, 151, 89*;* IR: 3306, 2917, 2850, 1738, 1534, 1261 cm^−1^; mp: 152–155 °C; *R*_f_: 0.40, (10% EtOAc, 90% hexane).

**(4(16)*Z*)-8-Hydroxy-6-nitrotricyclo[9.2.2.14,8]hexadeca-1(13),4,(16),6,11,14-pentaen-5-one (6) **^1^H NMR (500 MHz, DMSO-*d*_6_) δ (ppm) 7.48 (d, *J* = 8.1 Hz, 1H, H-13), 7.29 (d, *J* = 2.5 Hz, 1H, H-6), 7.16 (d, *J* = 8.1 Hz, 1H, H-12), 6.73 (d, *J* = 7.8 Hz, 1H, H-15), 6.58 (d, *J* = 7.7 Hz, 1H, H-16), 5.64–5.62 (m, 2H, H-8, OH), 2.94–2.90 (m, 1H, CH), 2.87–2.83 (dd, *J* = 8.3, 12.0 Hz, 1H, CH), 2.65–2.59 (m, 1H, CH), 2.53–2.52 (m, 1H, CH), 2.26–2.20 (m, 1H, CH), 2.02–1.93 (m, 2H, 2 × CH), 1.69–1.63 (m, 1H, CH); ^13^C NMR (126 MHz, DMSO-*d*_6_) δ (ppm) 177.3, 147.9, 147.4, 142.7, 141.1, 137.7, 130.6, 130.1, 129.3, 129.2, 68.2, 43.4, 33.4, 32.2, 30.2; HRMS-EI *m/z*: [M]^–^ calcd for C_16_H_15_NO_4_, 284.0917; found, 284.0928; ESIMS (*m/z*): [M + Na]^+^ 309, 287, 269, 240, 215, 194, 73*;* IR: 3453, 2925, 1665, 1535, 1356, 1010, 729 cm^−1^; mp: 206–213 °C; *R*_f_: 0.25 (20% EtOAc, 80% hexane).

**(4(16)*Z*)-8-Hydroxy-7-methoxy-6-nitrocyclo[9.2.2.14,8]hexadeca-1(13),4(16),11,14-tetraen-5-one (14) and (4(16)*Z*)-8-hydroxytricyclo[9.2.2.14,8]hexadeca-1(13),4(16),6,11,14-pentaen-5-one) (15):** To a solution of **6** (5.00 g, 17.5 mmol, 1.00 equiv) in CH_3_OH (0.20 M, 87.6 mL) was added TFA (1.00 M, 17.5 mL) and stirred at rt for 24 h. The solution was concentrated under reduced pressure yielding a light orange solid, which was purified by flash silica-gel chromatography (gradient of 0% to 60% EtOAc, hexane over 1 h) to give **6** (2.40 g, 8.41 mmol, 48%) and a mixture of **12** and **13**. Slow evaporation of fractions containing **12** and **13** initially gave clear crystals that could be separated to obtain pure characterization of **12** (1.95 g, 6.14 mmol, 35%) leaving behind **13**, which could be isolated as a light brown solid (0.38 g, 1.6 mmol, 9%).

**(4(16)*Z*)-8-Hydroxy-7-methoxy-6-nitrocyclo[9.2.2.14,8]hexadeca-1(13),4(16),11,14-tetraen-5-one (14) **^1^H NMR (500 MHz, CDCl_3_) δ (ppm) 7.45 (d, *J* = 8.2 Hz, 1H, H-13), 7.19 (d, *J* = 8.6 Hz, 1H, H-12), 7.05 (d, *J* = 8.0 Hz, 1H, H-15), 6.94 (d, *J* = 8.0 Hz, 1H, H-16), 5.61 (s, 1H, H-8), 5.17 (d, *J* = 10.8 Hz, 1H, H-5), 4.40 (d, *J* = 11.0 Hz, 1H, H-6), 3.62 (s, 3H, H-17), 3.17 (dd, *J* = 6.6, 14.1 Hz, 1H, CH), 2.94−2.86 (m, 2H, 2 × CH), 2.58−2.47 (m, 2H, 2 × CH), 1.99 (dd, *J* = 5.7, 14.4 Hz, 1H, CH_2_), 1.90 (dt, *J* = 6.7, 13.1 Hz, 1H, CH), 1.68−1.61 (m, 1H, CH); ^13^C NMR (126 MHz, CDCl_3_) δ (ppm) 187.3, 145.2, 143.6, 132.0, 136.1, 130.8, 130.7, 130.5, 130.0, 92.7, 75.5, 72.0, 61.3, 42.3, 35.1, 33.3, 31.7; HRMS-EI *m*/*z*: [M + Na]^+^ calcd for C_16_H_19_NO_5_Na, 340.1152; found, 340.1172; ESIMS (*m/z*): [M]^–^ 316, 284, 255, 205, 145, 97; IR: 3525, 3455, 2934, 1683, 1557, 1367, 1074, 1056, 729 cm^–1^; mp: 209–212 °C; *R*_f_: 0.25 (30% EtOAc, 70% hexane).

**(4(16)*Z*)-8-Hydroxytricyclo[9.2.2.14,8]hexadeca-1(13),4(16),6,11,14-pentaen-5-one) (15) **^1^H NMR (700 MHz, CDCl_3_) δ (ppm) 7.36 (d, *J* = 8.1 Hz, 1H, H-13), 7.09 (dd, *J* = 1.2, 8.2 Hz, 1H, H-12), 6.69 (AB q, *J* = 1.5, 8.0 Hz, 2H, H-15, H-16), 6.42 (dd, *J* = 2.8, 9.9 Hz, 1H, H-5), 6.02 (d, *J* = 9.9 Hz, 1H, H-6), 5.48 (d, *J* = 2.9 Hz, 1H, H-8), 3.03–3.00 (m, 1H, CH), 2.86 (dd, *J* = 4.1, 9.5 Hz, 1H, CH), 2.70 (dt, *J* = 3.9, 12.5 Hz, 1H, CH), 2.27-2.22 (m, 1H, CH), 1.99–1.96 (m, 1H, CH), 1.54–1.49 (m, 3H, 3 × CH); ^13^C NMR (176 MHz, CDCl_3_) δ (ppm) 187.2, 145.8, 145.6, 143.4, 137.0, 132.1, 131.0, 129.9, 129.3, 129.1, 69.6, 42.9, 34.5, 33.1, 31.1; ^1^H NMR (700 MHz, acetone-*d*_6_) δ (ppm) 7.45 (d, *J* = 7.9 Hz, 1H, H-13), 7.15 (dd, *J* = 1.6, 7.7 Hz, 1H, H-12), 6.70 (dd, *J* = 1.6, 7.7 Hz, 1H, H-15), 6.56 (dd, *J* = 1.7, 7.7 Hz, 1H, H-16), 6.47 (dd, *J* = 2.9, 10.0 Hz, 1H, H-5), 5.87 (d, *J* = 10.0 Hz, 1H, H-6), 5.48 (d, *J* = 2.8 Hz, 1H, H-8), 2.96 (ddd, *J* = 2.7, 5.6, 13.8 Hz, 1H, CH), 2.73 (dd, *J* = 7.4, 10.5 Hz, 1H, CH), 2.70 (dd, *J* = 7.3, 10.6 Hz, 1H, CH), 2.57 (dd, *J* = 7.4, 12.4 Hz, 1H, CH), 2.26 (ddd, *J* = 6.5, 12.2, 13.6 Hz, 1H, CH), 2.00–1.93 (m, 3H, 3 × CH); ^13^C NMR (176 MHz, acetone-*d*_6_) δ (ppm) 186.8, 147.0, 146.6, 143.1, 137.7, 130.7, 130.0, 198.8, 194.4, 128.3, 128.3, 68.6, 43.2, 34.1, 32.8, 30.8; HRMS-EI *m*/*z*: [M + Na]^+^ calcd for C_16_H_16_O_2_Na, 263.1043; found, 263.1054; ESIMS (*m/z*): [M]^+^ 241, 224, 118, 104; IR: 3386, 2918, 2849, 1651, 1622, 1261, 1020, 829, 813 cm^−1^; mp: 206–208 °C; *R*_f_: 0.25 (30% EtOAc, 70% hexane).

## Supporting Information

File 1 Metaparacyclophane spectra.
