# Supplementary material for: Breaking paracyclophane: the unexpected formation of non-symmetric disubstituted nitro[2.2]metaparacyclophanes
Source: Beilstein J Org Chem. 2021 Jun 29;17:1518–26. doi: 10.3762/bjoc.17.109 (PMC8261525; doi:10.3762/bjoc.17.109)

BEILSTEIN JOURNAL OF ORGANIC CHEMISTRY

Supporting Information

for

Breaking paracyclophane: the unexpected formation of non-symmetric disubstituted nitro[2.2]metaparacyclophanes

Suraj Patel, Tyson N. Dais, Paul G. Plieger and Gareth J. Rowlands

Beilstein J. Org. Chem. **2021,** *17,* 1518–1526. doi:10.3762/bjoc.17.109

Metaparacyclophane spectra

License and Terms: This is a supporting information file under the terms of the Creative Commons Attribution License (https://creativecommons.org/licenses/by/4.0). Please note that the reuse, redistribution and reproduction in particular requires that the author(s) and source are credited and that individual graphics may be subject to special legal provisions.
The license is subject to the *Beilstein Journal of Organic Chemistry* terms and conditions: (https://www.beilstein-journals.org/bjoc/terms)

^{1}H NMR spectrum of (±)-**5** in $CDCl_3$

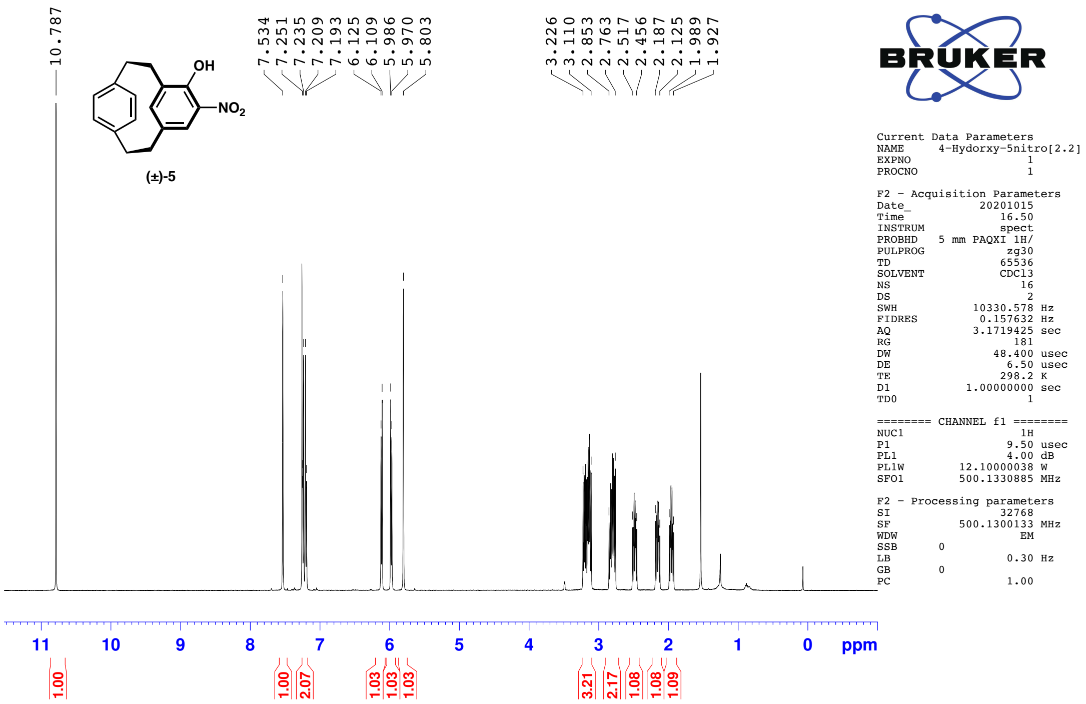

^{13}C NMR spectrum of (±)-**5** in $CDCl_3$

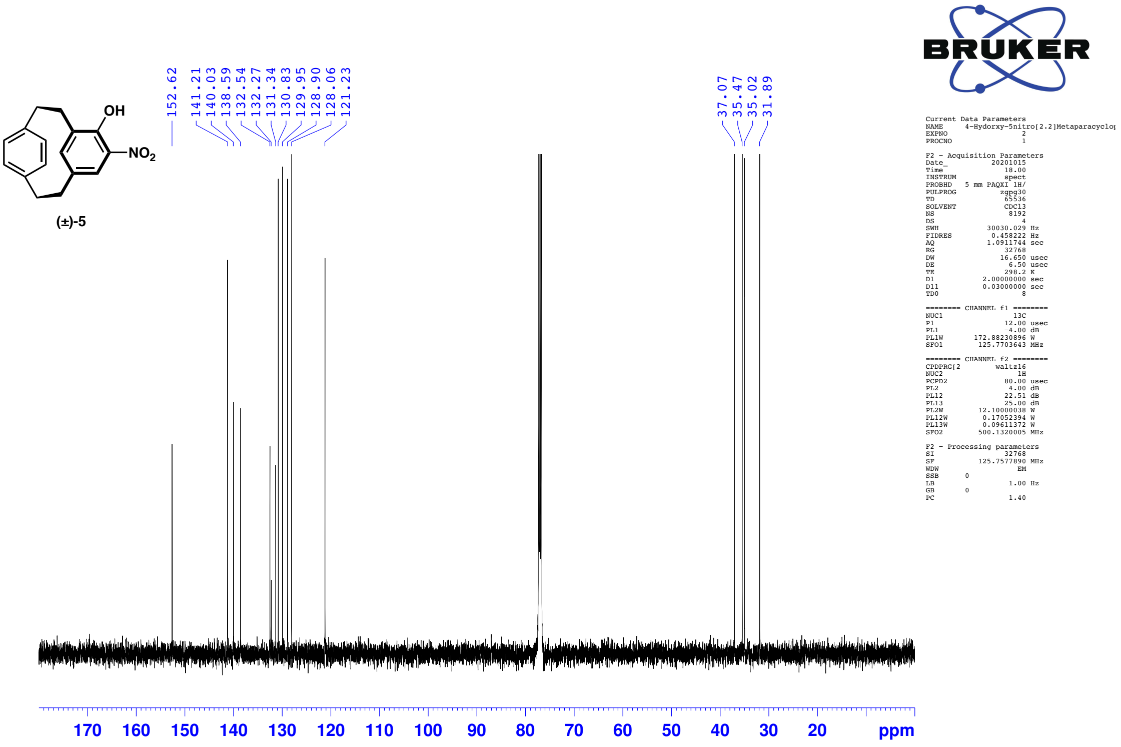

DEPT NMR spectrum of (±)-**5** in $CDCl_3$

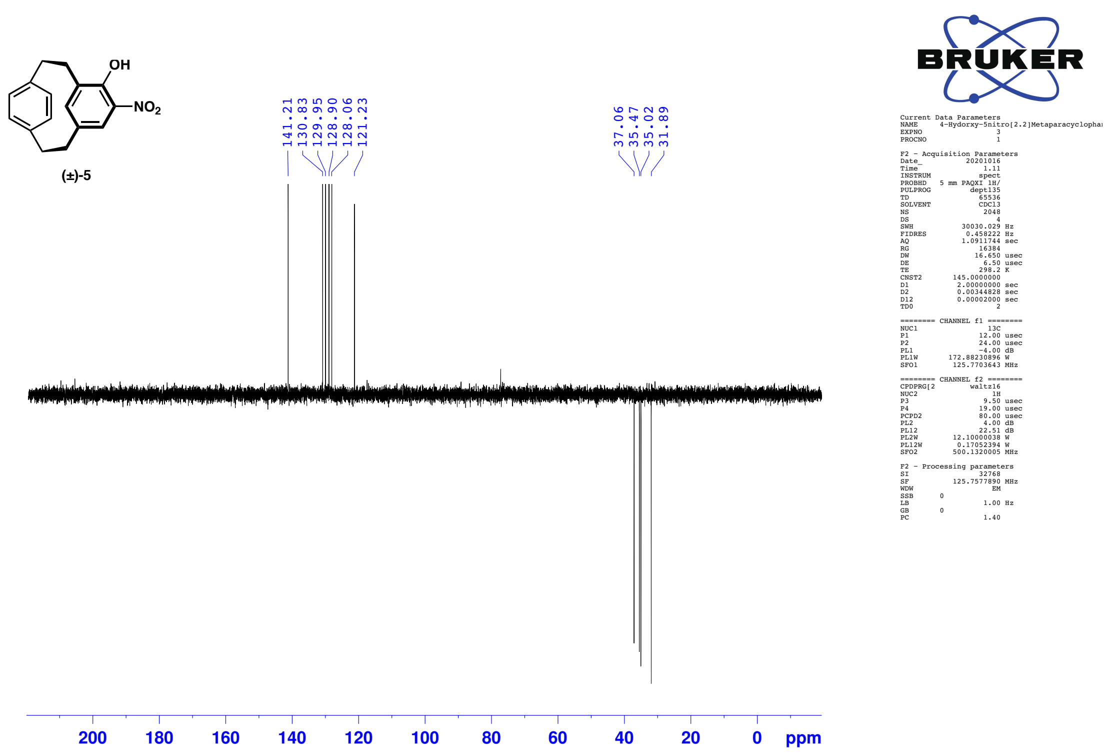

NOESY spectrum of (±)-**5** in $CDCl_3$

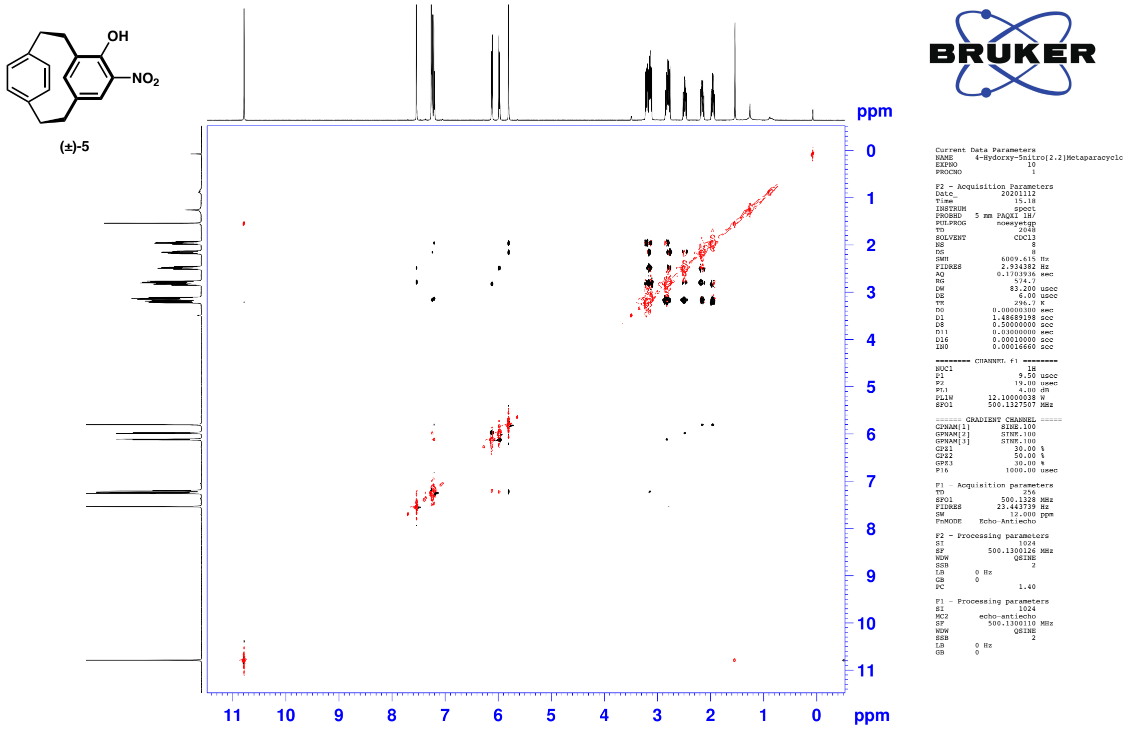

COSY NMR spectrum of (±)-**5** in $CDCl_3$

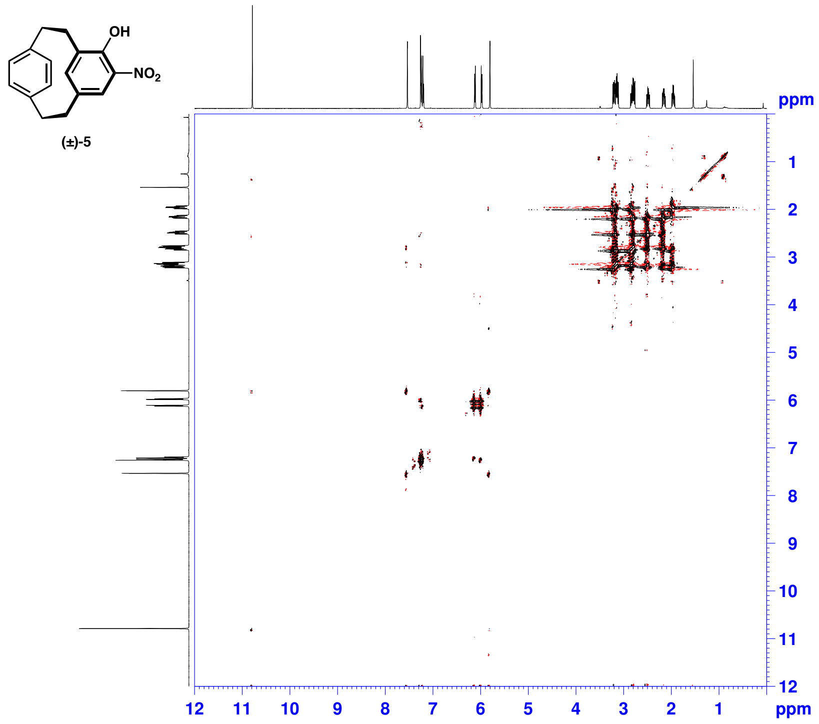


```
Current Data Parameters
NAME      4-Hydorxy-5nitro[2.2]Metaparacyclophar
EXPNO                  4
PROCNO                 1

F2 - Acquisition Parameters
Date_           20201016
Time                2.05
INSTRUM            spect
PROBHD   5 mm PAQXI 1H/
PULPROG       cosygpmfph
TD                  2048
SOLVENT            CDCl3
NS                     8
DS                     4
SWH             6510.417 Hz
FIDRES          3.178914 Hz
AQ             0.1572864 sec
RG                   256
DW                76.800 usec
DE                  6.50 usec
TE                 298.2 K
D0            0.00006481 sec
D1            2.00000000 sec
D13           0.00000400 sec
D16           0.00010000 sec
IN0           0.00015380 sec

======== CHANNEL f1 ========
NUC1                  1H
P1                  9.50 usec
P2                 19.00 usec
PL1                 4.00 dB
PL1W         12.10000038 W
SFO1         500.1327507 MHz

====== GRADIENT CHANNEL =====
GPNAM[1]        SINE.100
GPNAM[2]        SINE.100
GPZ1               10.00 %
GPZ2               20.00 %
P16              1000.00 usec

F1 - Acquisition parameters
TD                   400
SFO1            500.1328 MHz
FIDRES         16.254314 Hz
SW                13.000 ppm
FnMODE       States-TPPI

F2 - Processing parameters
SI                  1024
SF           500.1300000 MHz
WDW                QSINE
SSB                    2
LB        0 Hz
GB        0
PC                  1.40

F1 - Processing parameters
SI                  1024
MC2          States-TPPI
SF           500.1300000 MHz
WDW                QSINE
SSB                    2
LB        0 Hz
GB        0
```

Long range COSY NMR spectrum of (±)-**5** in $CDCl_3$

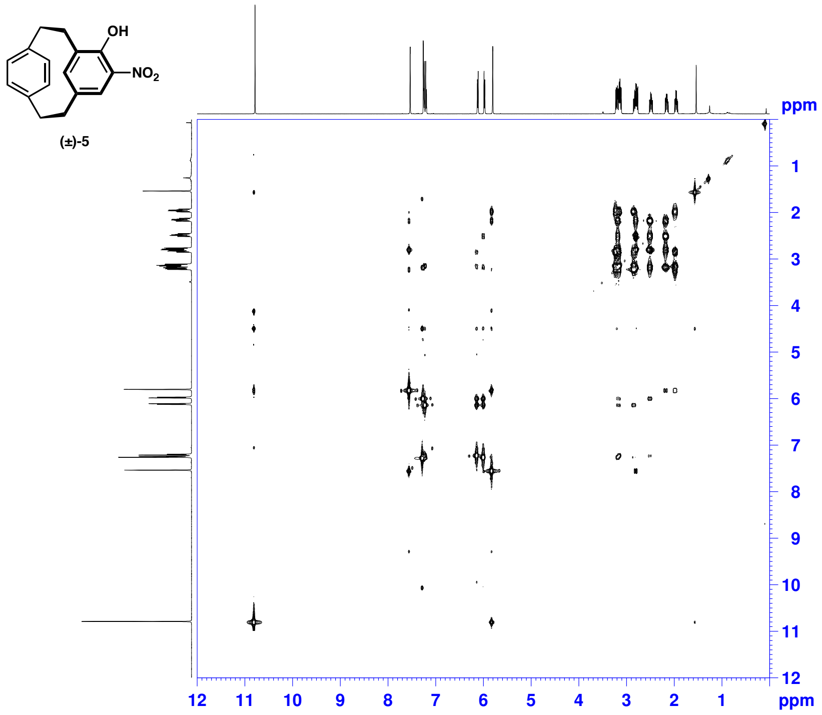


```
Current Data Parameters
NAME      4-Hydorxy-5nitro[2.2]Metaparacyclophane
EXPNO                  5
PROCNO                 1

F2 - Acquisition Parameters
Date_           20201016
Time                4.01
INSTRUM            spect
PROBHD   5 mm PAQXI 1H/
PULPROG         cosylrqf
TD                  2048
SOLVENT            CDCl3
NS                     8
DS                     4
SWH             6510.417 Hz
FIDRES          3.178914 Hz
AQ             0.1572864 sec
RG                  1024
DW                76.800 usec
DE                  5.50 usec
TE                 298.2 K
D0            0.00000300 sec
D1            2.00000000 sec
D6            0.20000000 sec
IN0           0.00015380 sec

======== CHANNEL f1 ========
NUC1                  1H
P1                  9.50 usec
PL1                 4.00 dB
PL1W         12.10000038 W
SFO1         500.1322506 MHz

F1 - Acquisition parameters
TD                   400
SFO1            500.1323 MHz
FIDRES         16.254297 Hz
SW                13.000 ppm
FnMODE                QF

F2 - Processing parameters
SI                  1024
SF           500.1300000 MHz
WDW                 SINE
SSB       0
LB        0 Hz
GB        0
PC                  1.40

F1 - Processing parameters
SI                  1024
MC2                   QF
SF           500.1300000 MHz
WDW                 SINE
SSB       0
LB        0 Hz
GB        0
```

HMQC NMR spectrum of (±)-**5** in $CDCl_3$

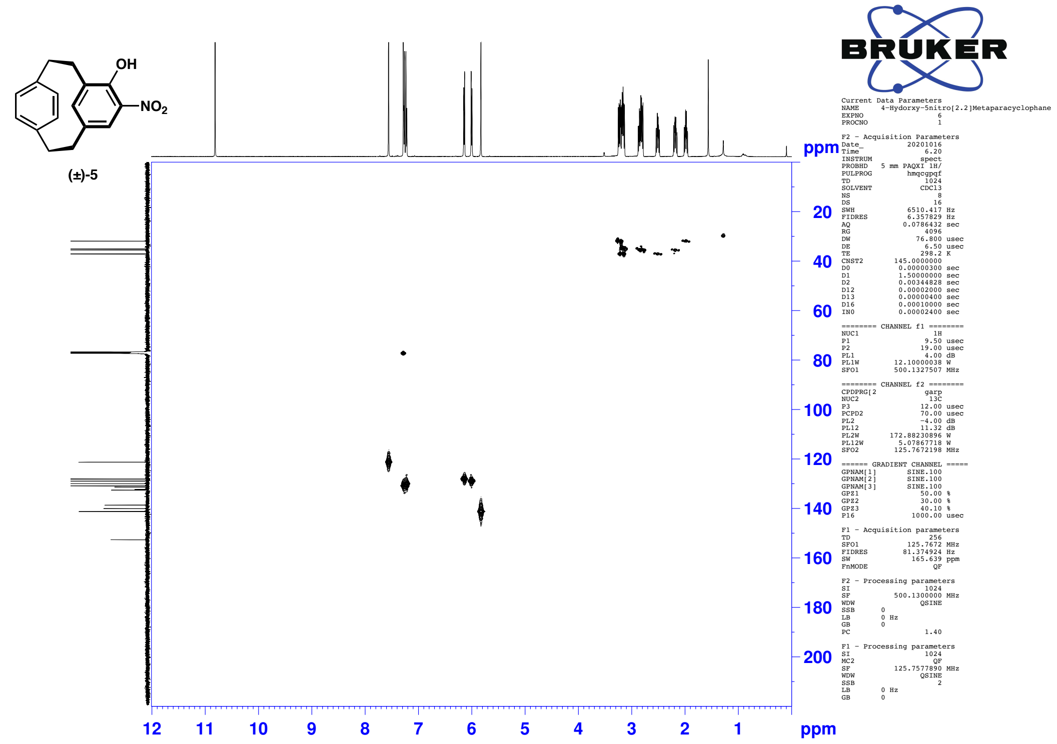

HMBC spectrum of (±)-**5** in $CDCl_3$

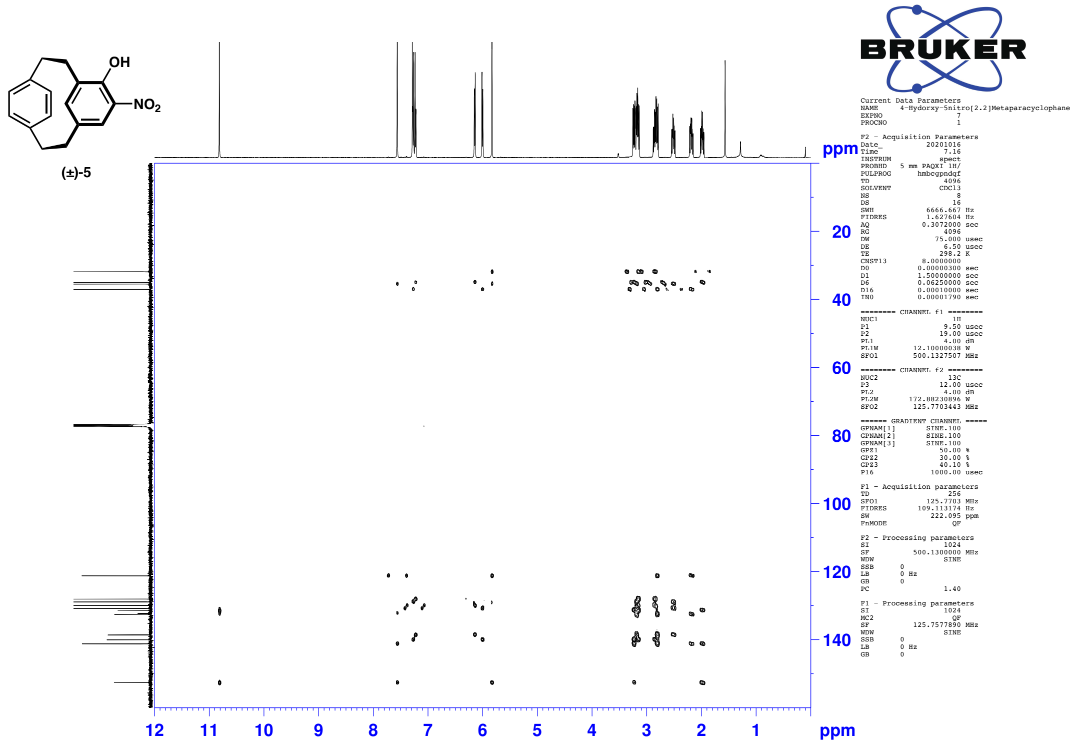

^{1}H NMR spectrum of **6** in DMSO-d_6

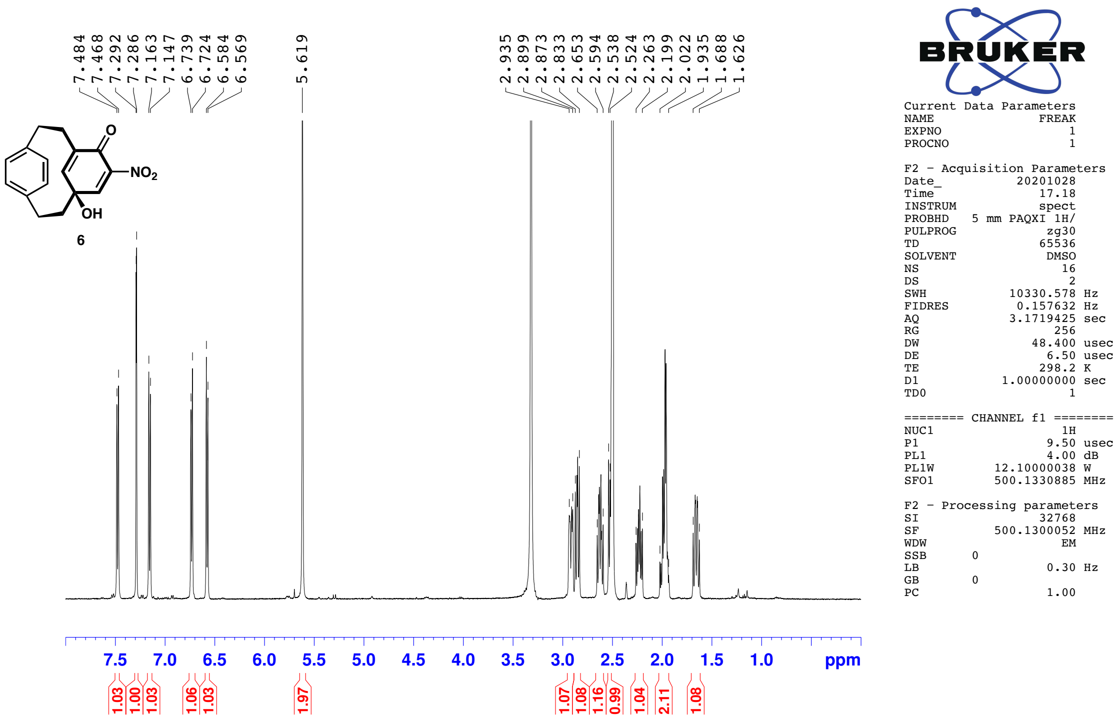

^{13}C NMR spectrum of **6** in DMSO-d_6

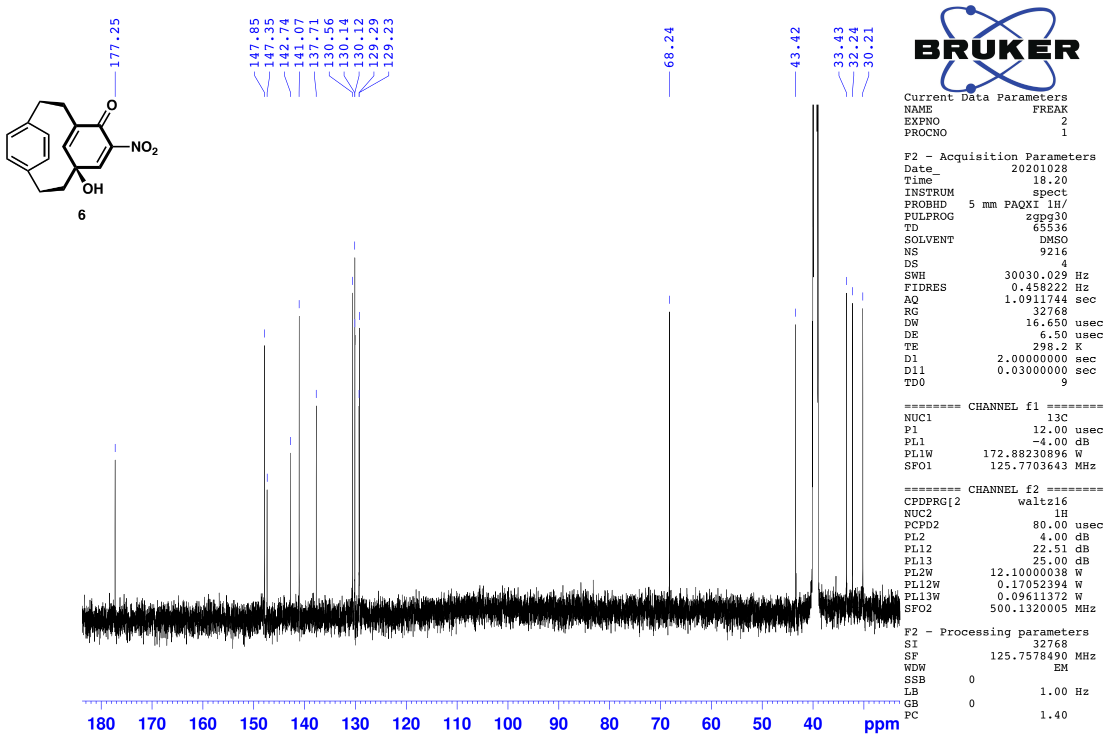

DEPT NMR spectrum of **6** in DMSO-d_6

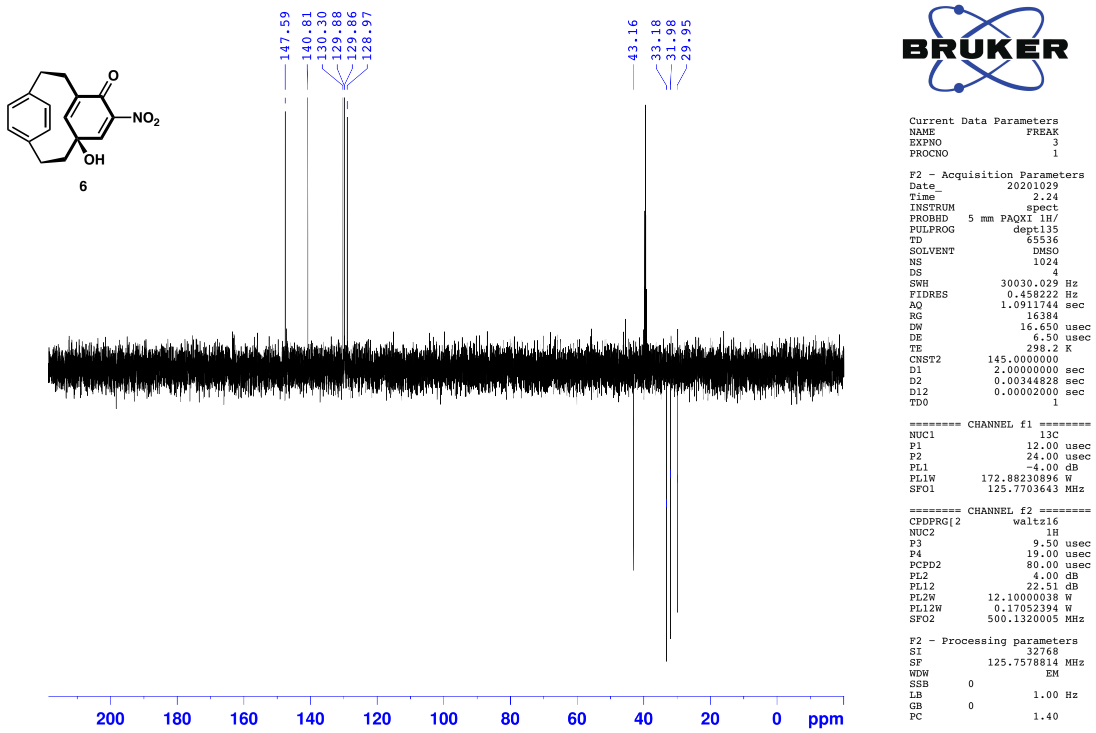

NOESY spectrum of **6** in DMSO-d_6

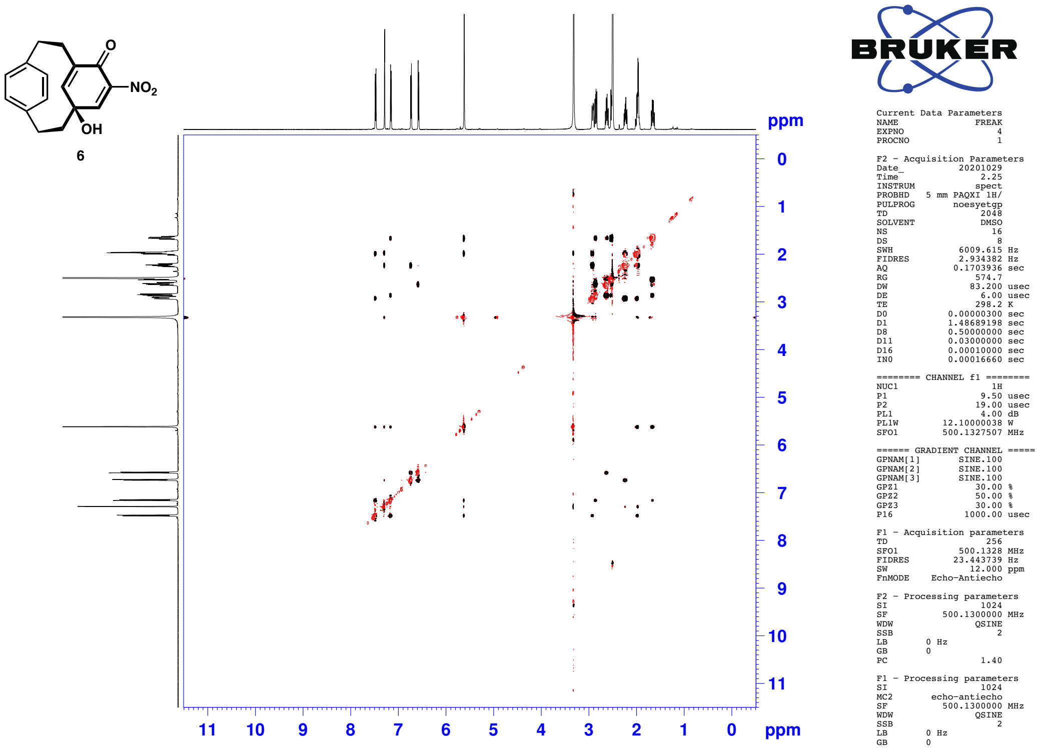

COSY NMR spectrum of **6** in DMSO-d_6

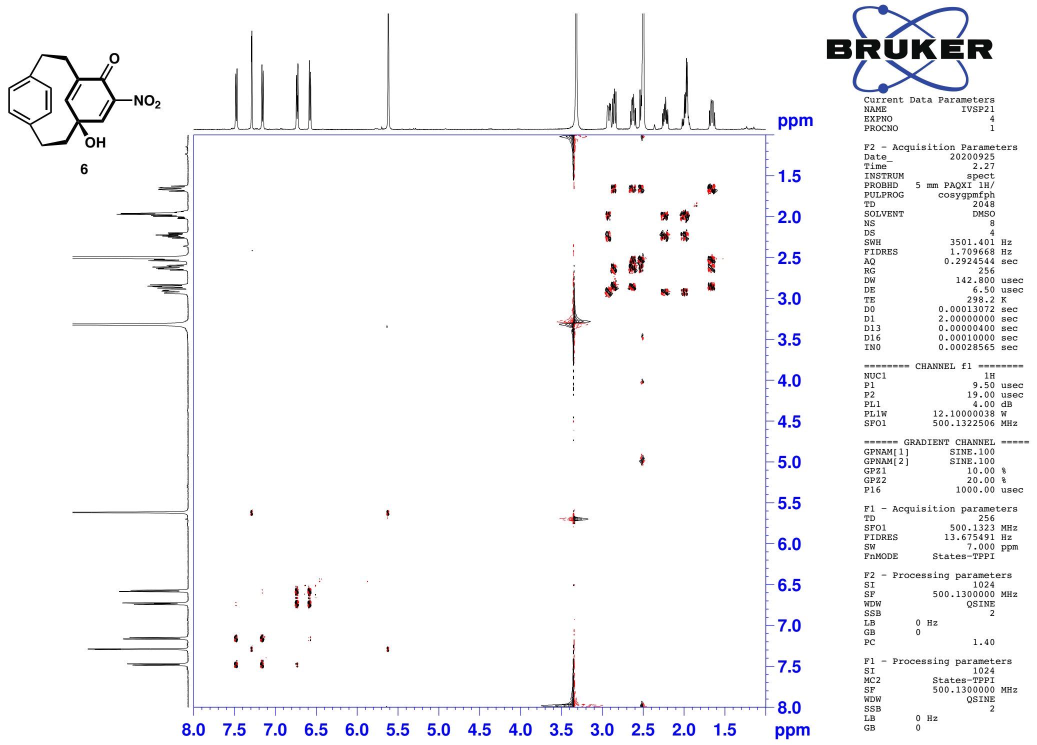

Long range COSY NMR spectrum of **6** in DMSO-d_6

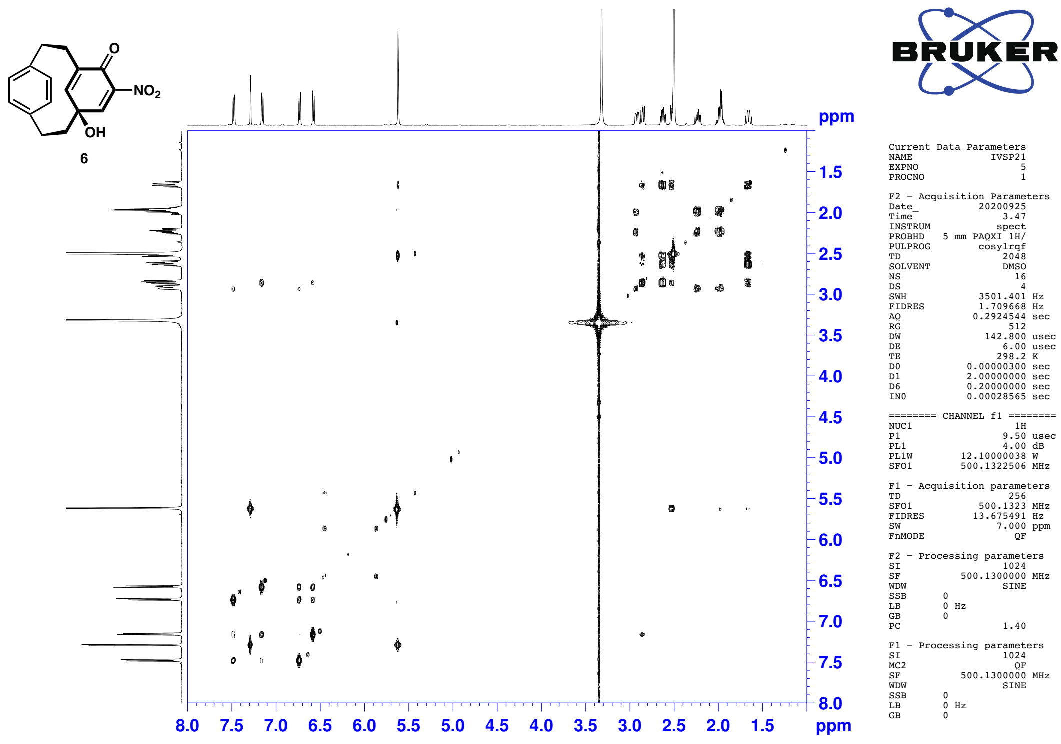

HMQC NMR spectrum of **6** in DMSO-d_6

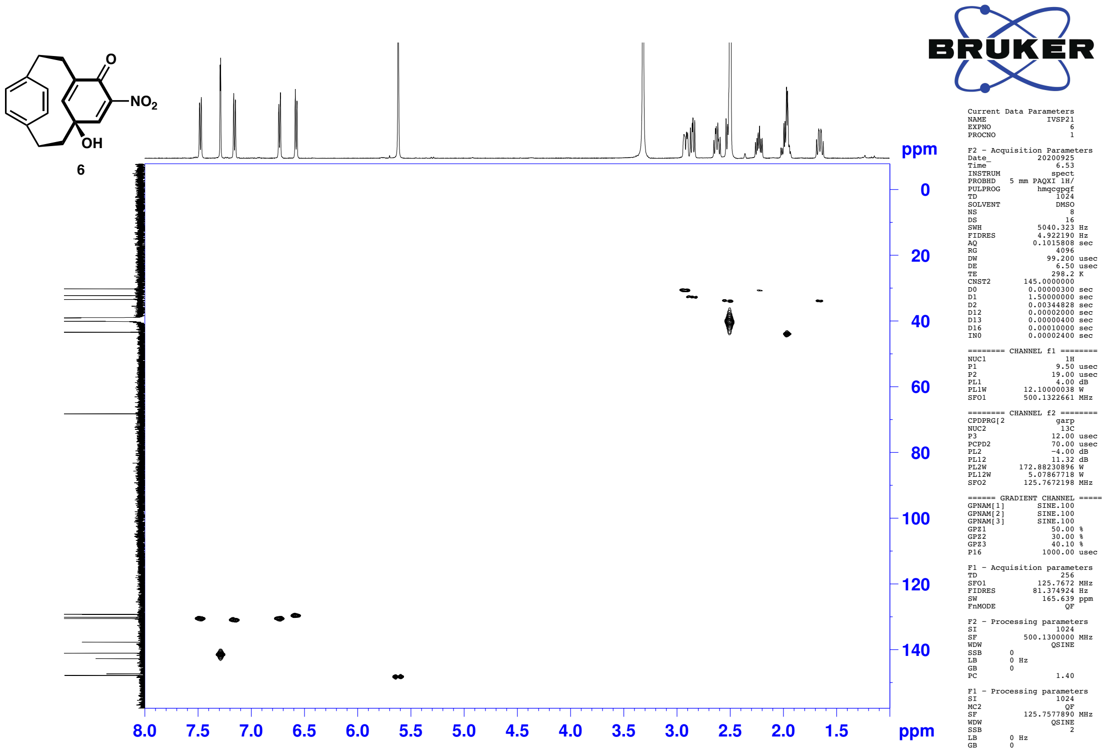

HMBC NMR spectrum of **6** in DMSO-d_6

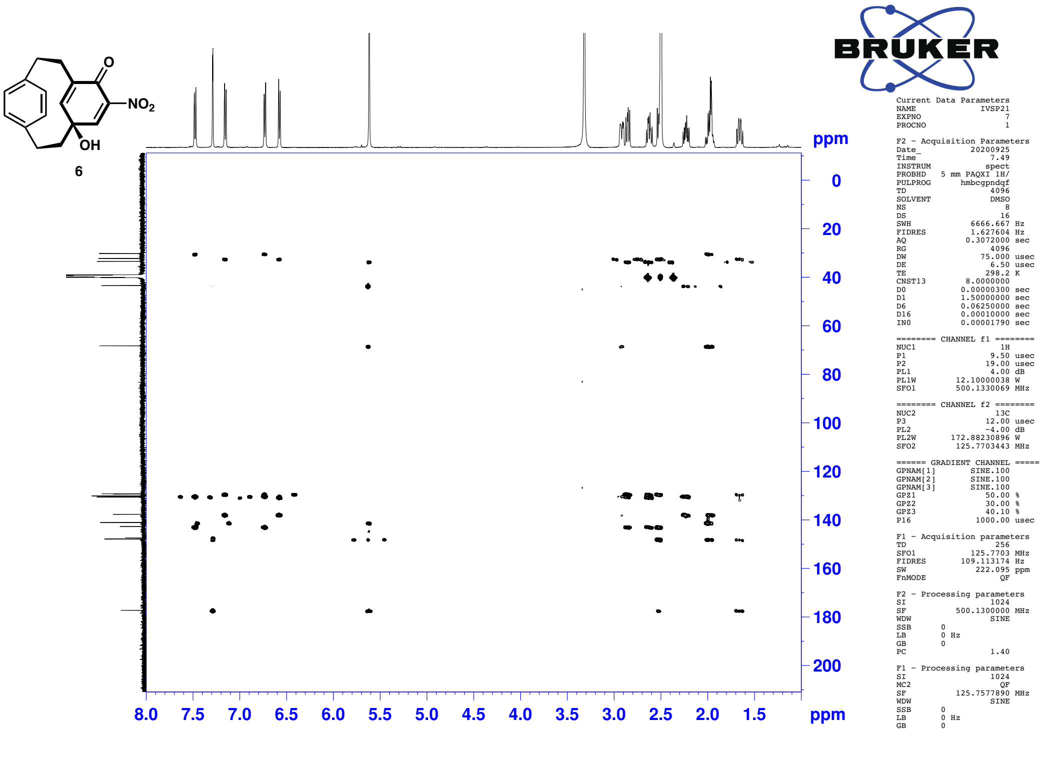

^1H NMR spectrum of **14** in $CDCl_3$

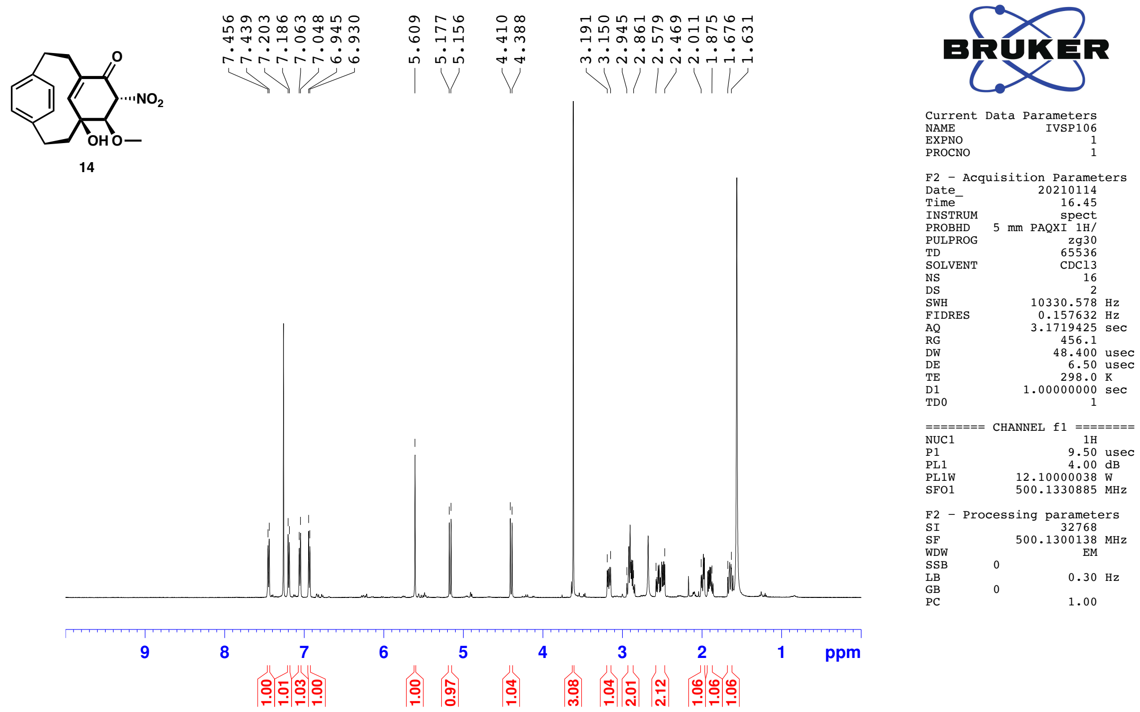

^{13}C NMR spectrum of **14** in $CDCl_3$

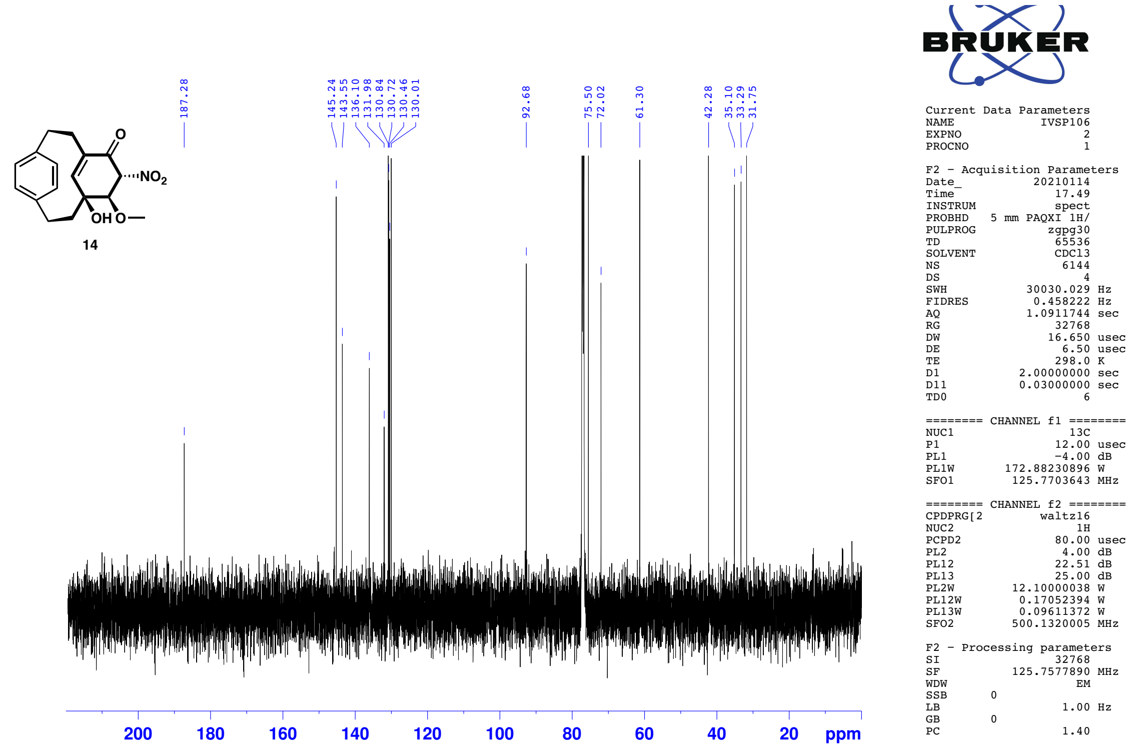

DEPT spectrum of **14** in $CDCl_3$

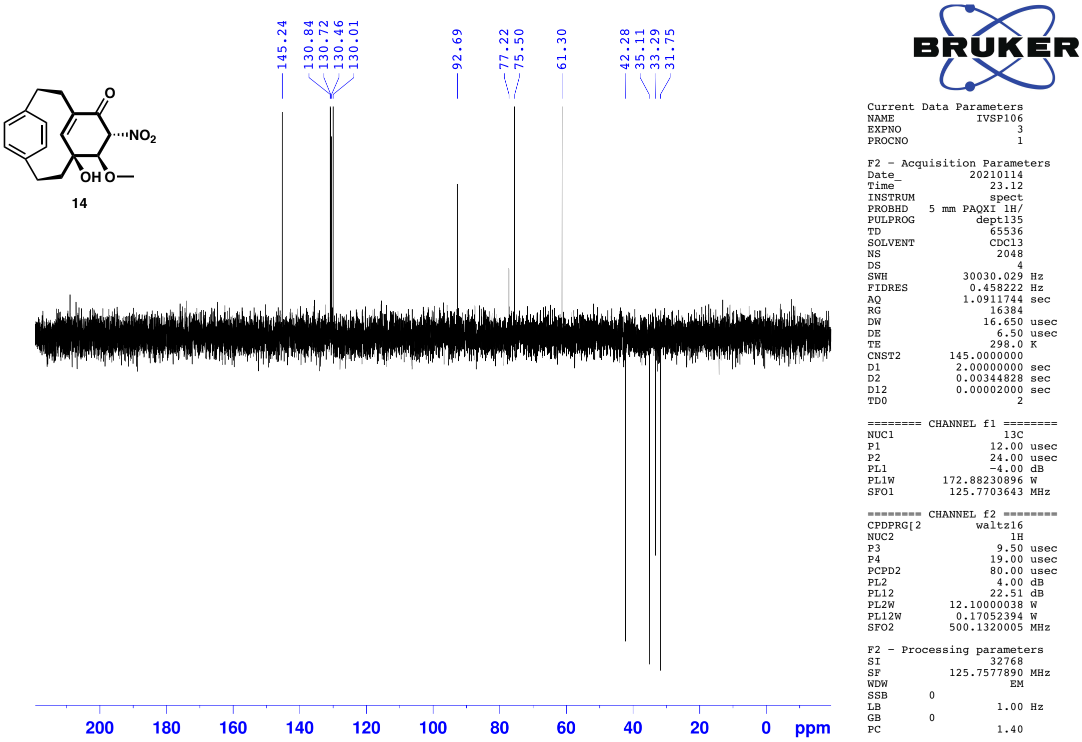

NOESY spectrum of **14** in $CDCl_3$

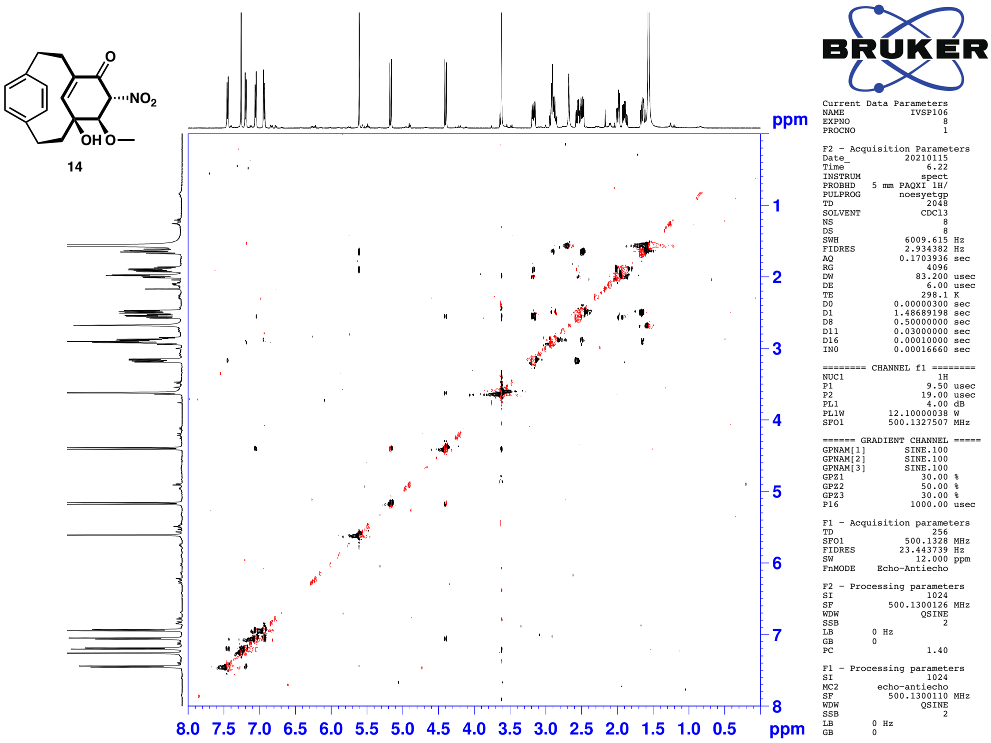

COSY spectrum of **14** in $CDCl_3$

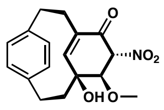

14

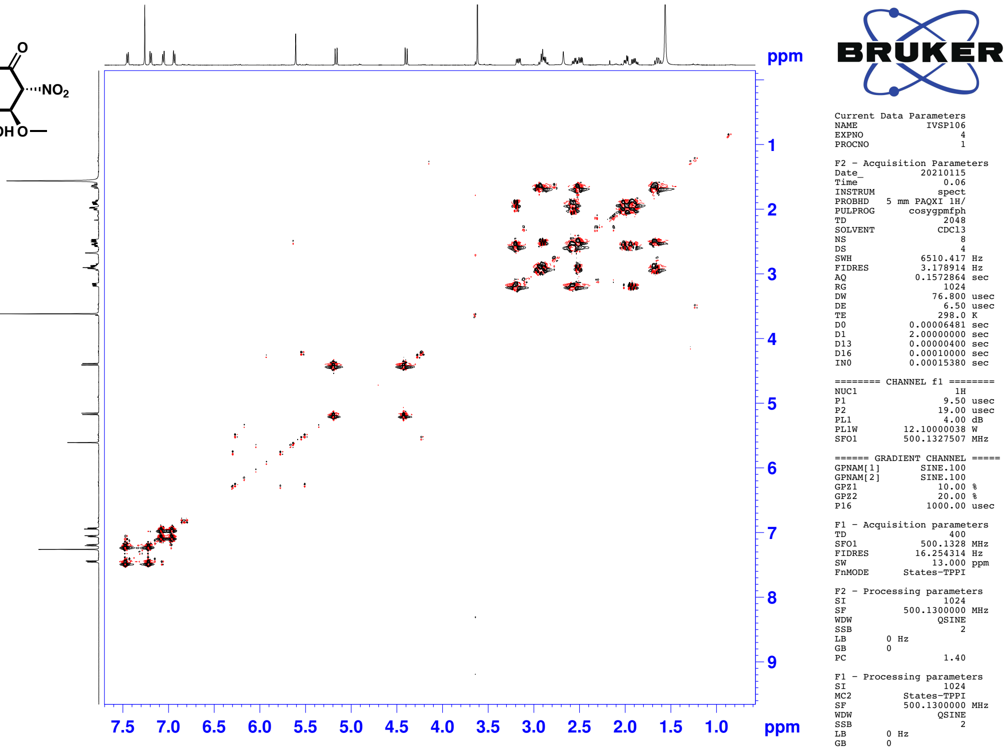

Long range COSY spectrum of **14** in $CDCl_3$

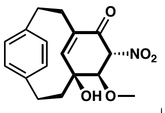

14

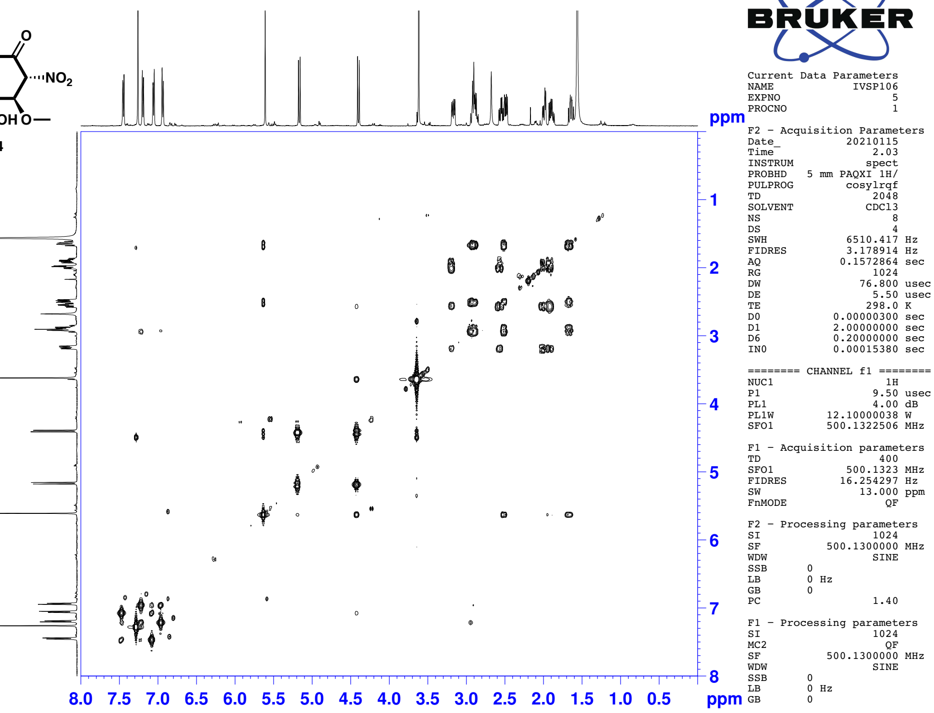

HMQC spectrum of **14** in $CDCl_3$

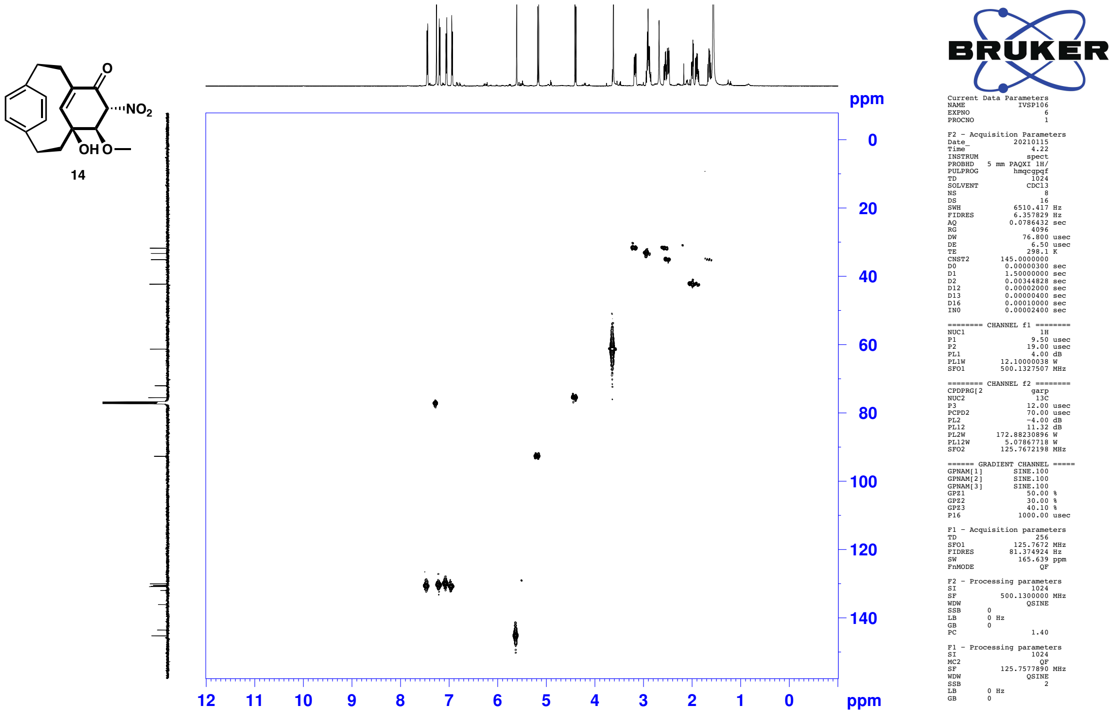

HMBC spectrum of **14** in $CDCl_3$

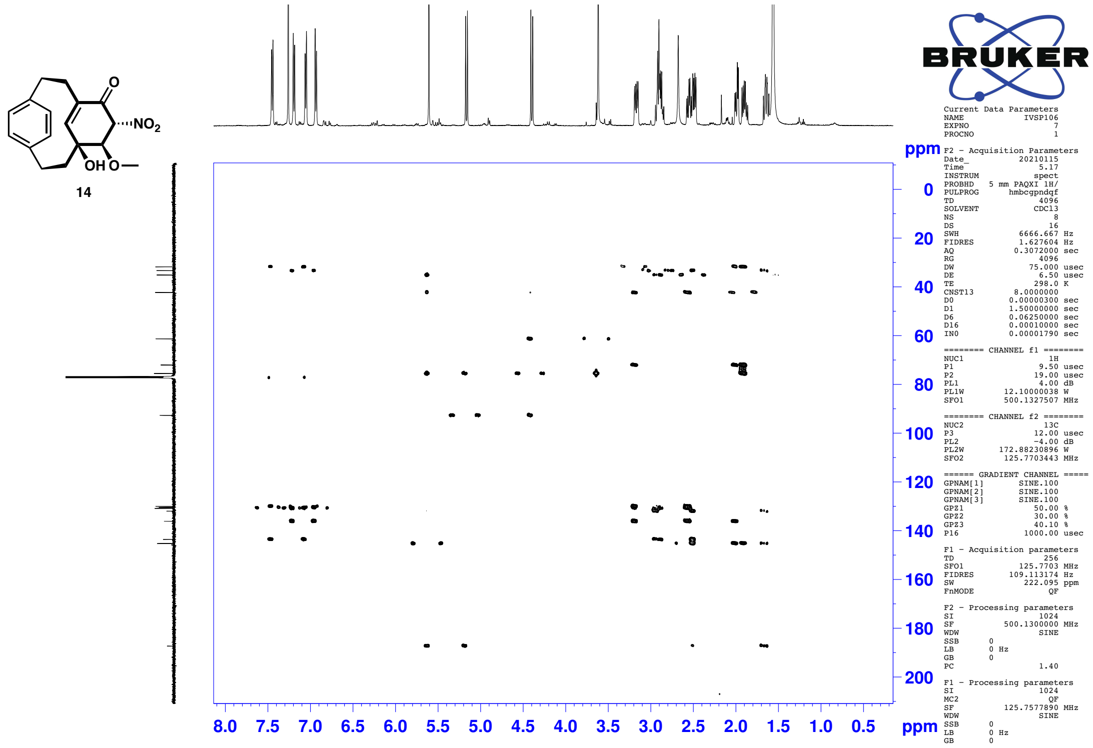

^{1}H NMR spectrum of **15** in $CDCl_3$

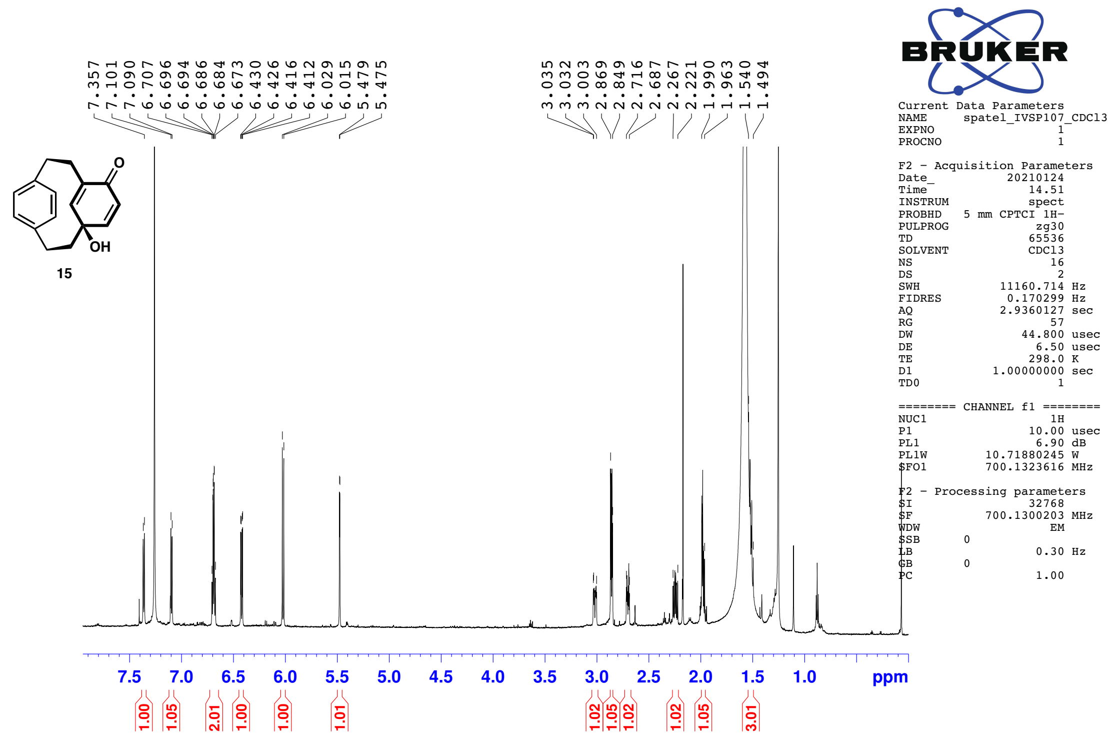

^{13}C NMR spectrum of **15** in $CDCl_3$

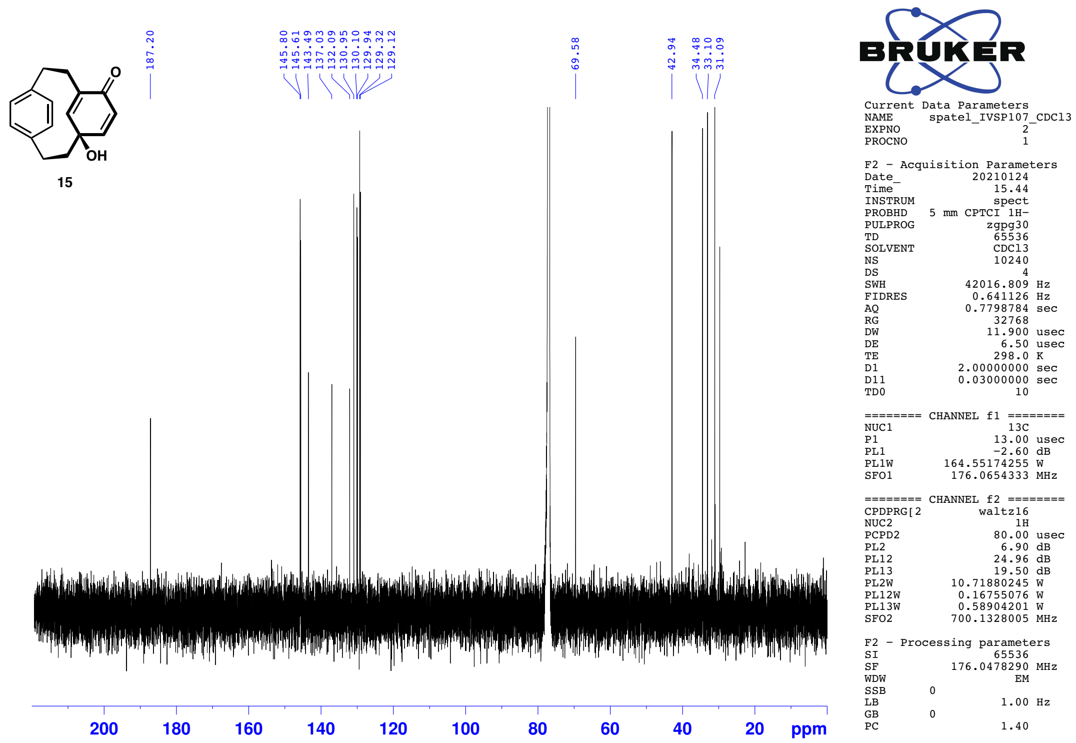

DEPT spectrum of **15** in $CDCl_3$

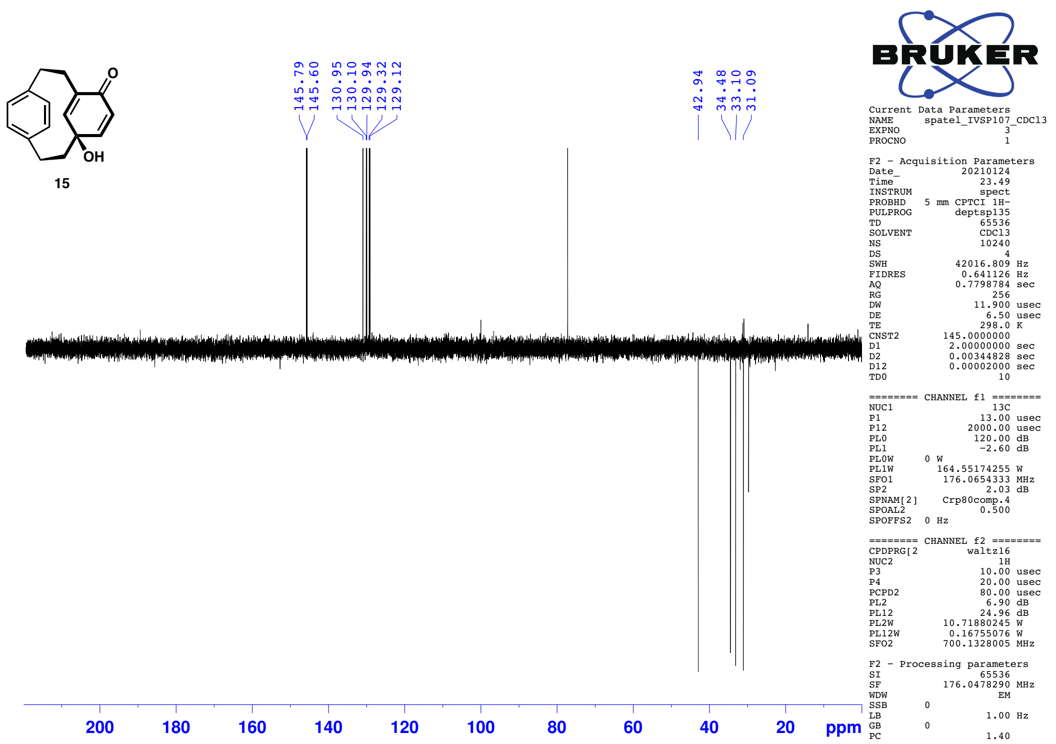

NOESY spectrum of **15** in $CDCl_3$

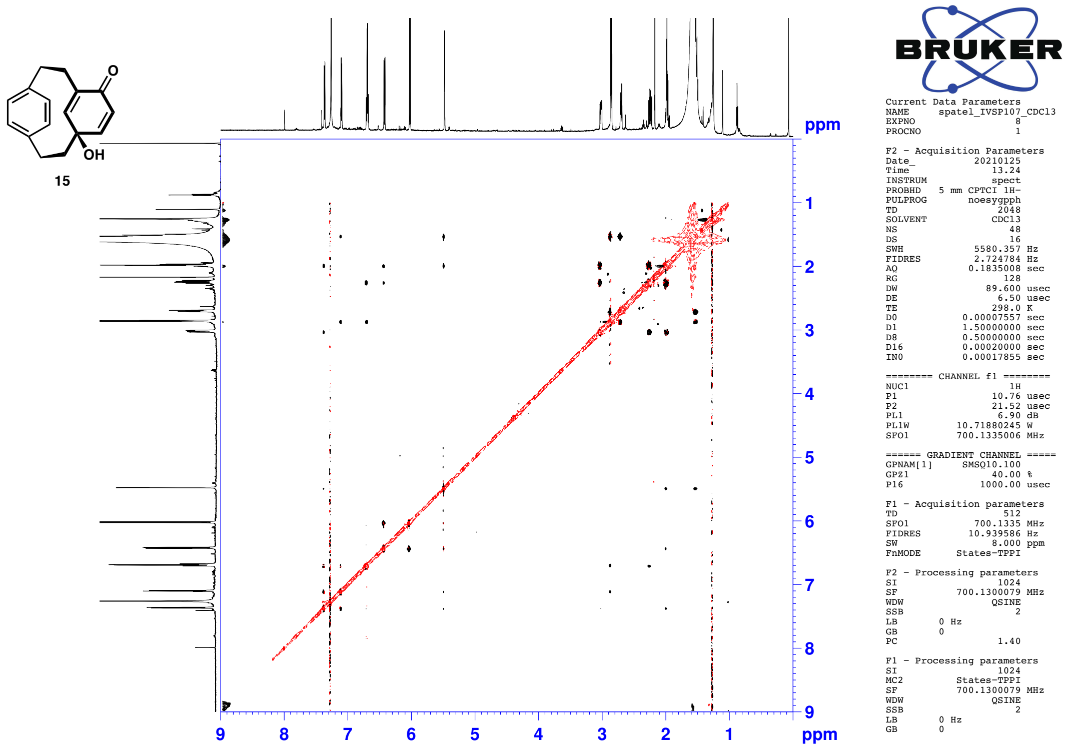

COSY spectrum of **15** in $CDCl_3$

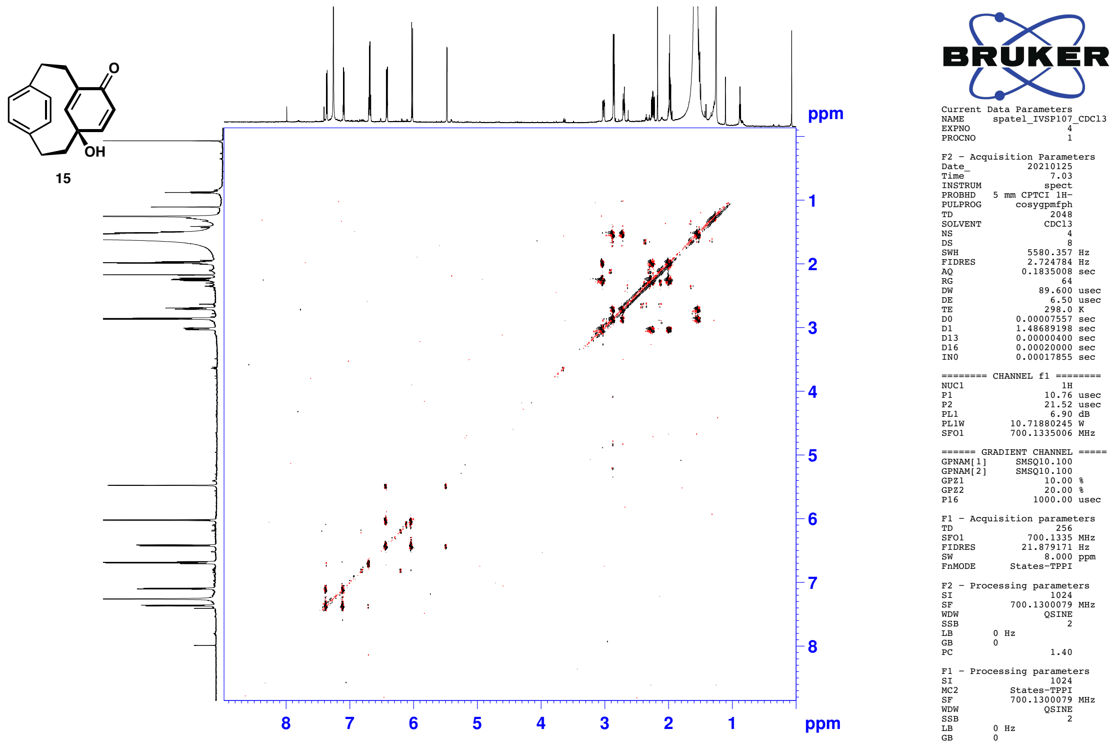

Long range COSY spectrum of **15** in $CDCl_3$

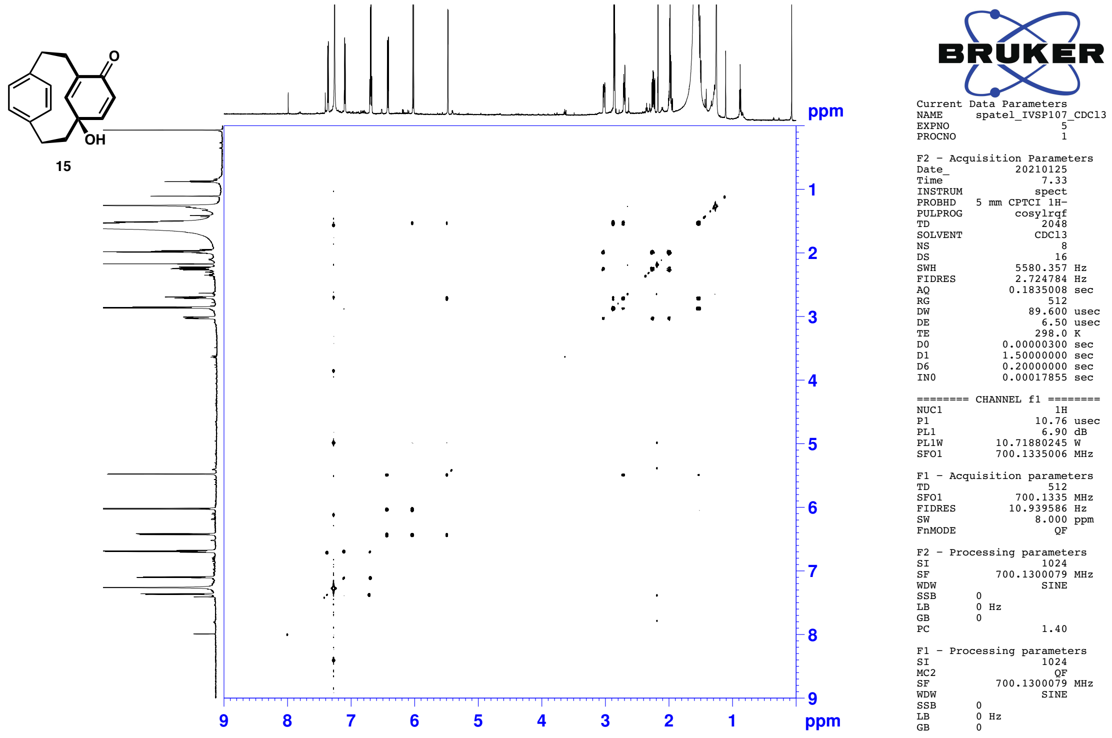

HMQC spectrum of **15** in $CDCl_3$

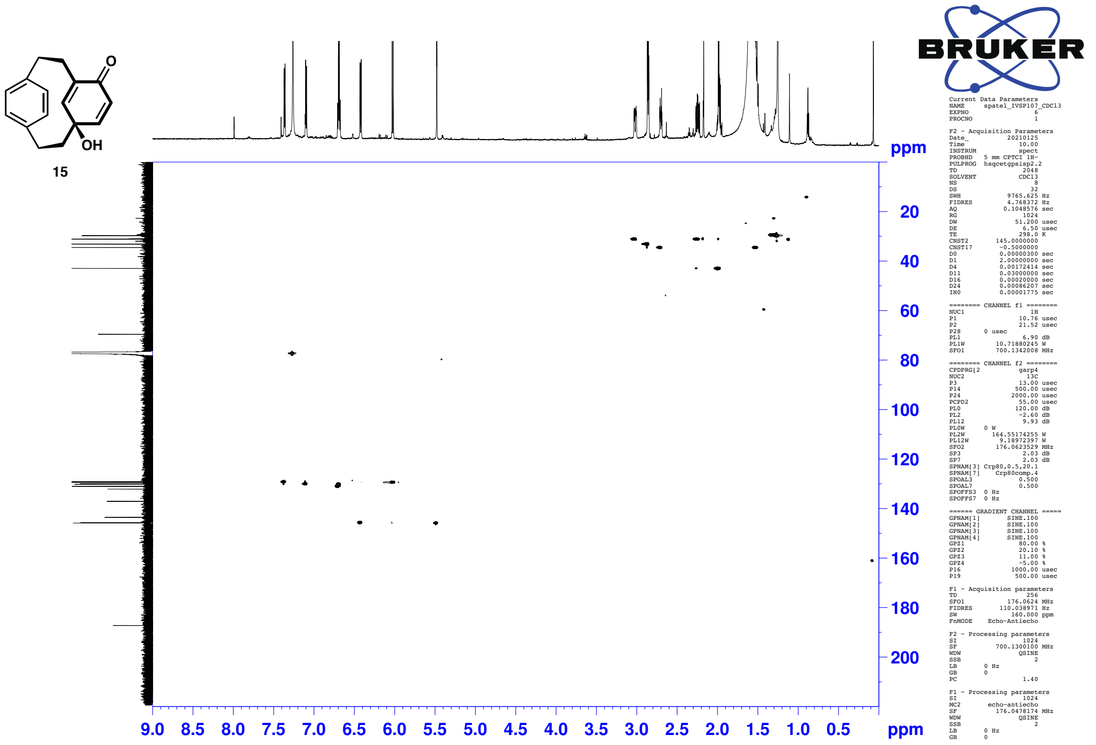

HMBC spectrum of **15** in $CDCl_3$

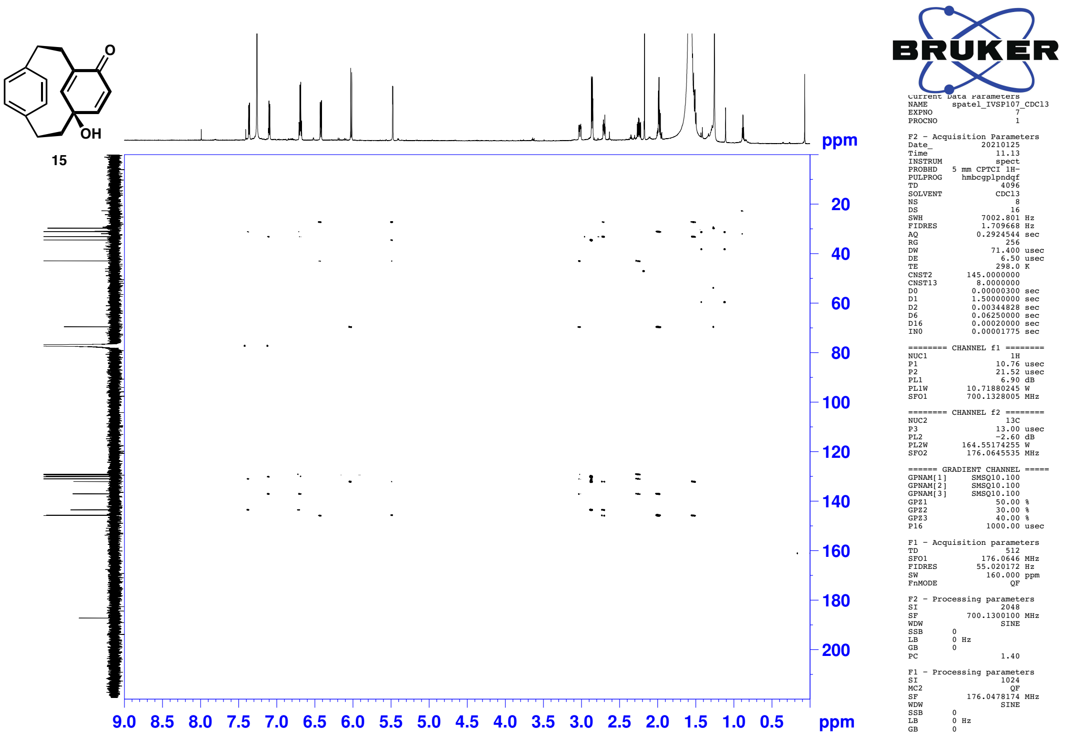

^{1}H NMR spectrum of **15** in $(CD_3)_2CO$

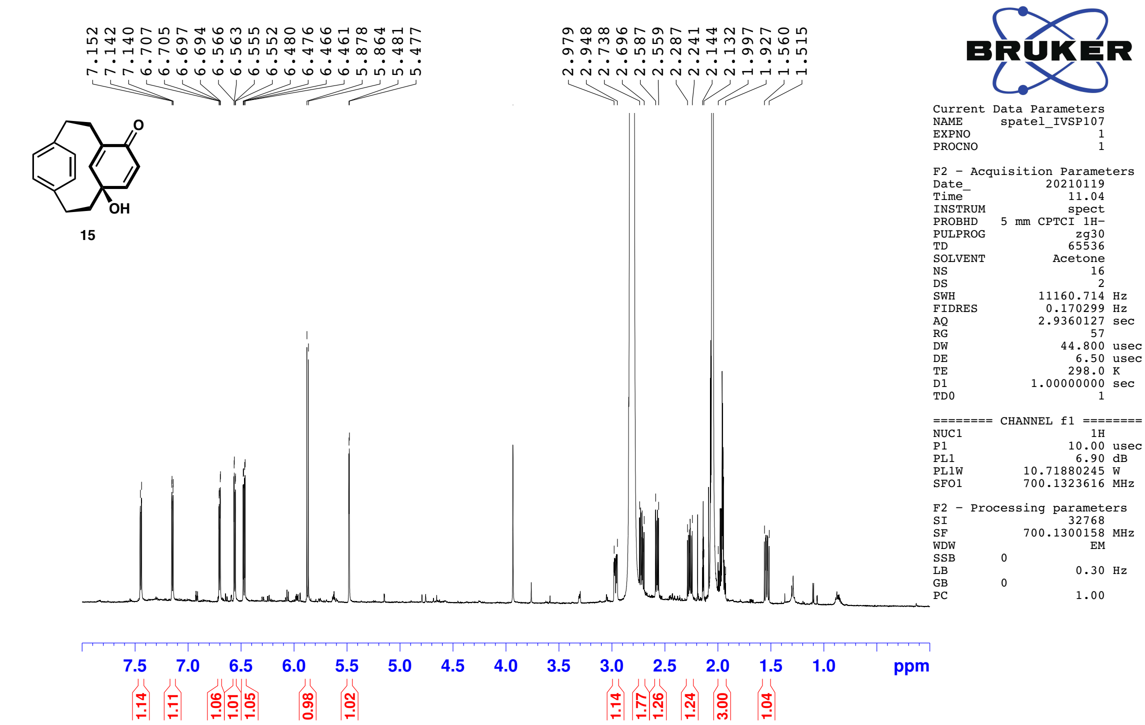

^{13}C NMR spectrum of **15** in $(CD_3)_2CO$

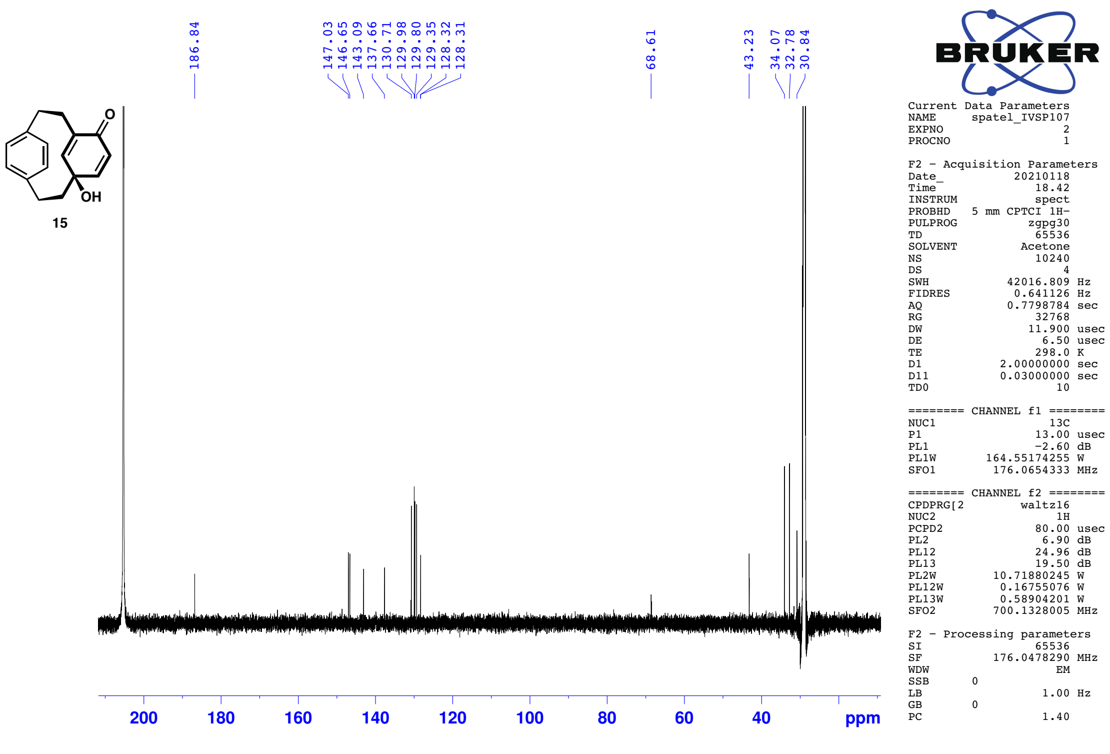

DEPT spectrum of **15** in $(CD_3)_2CO$

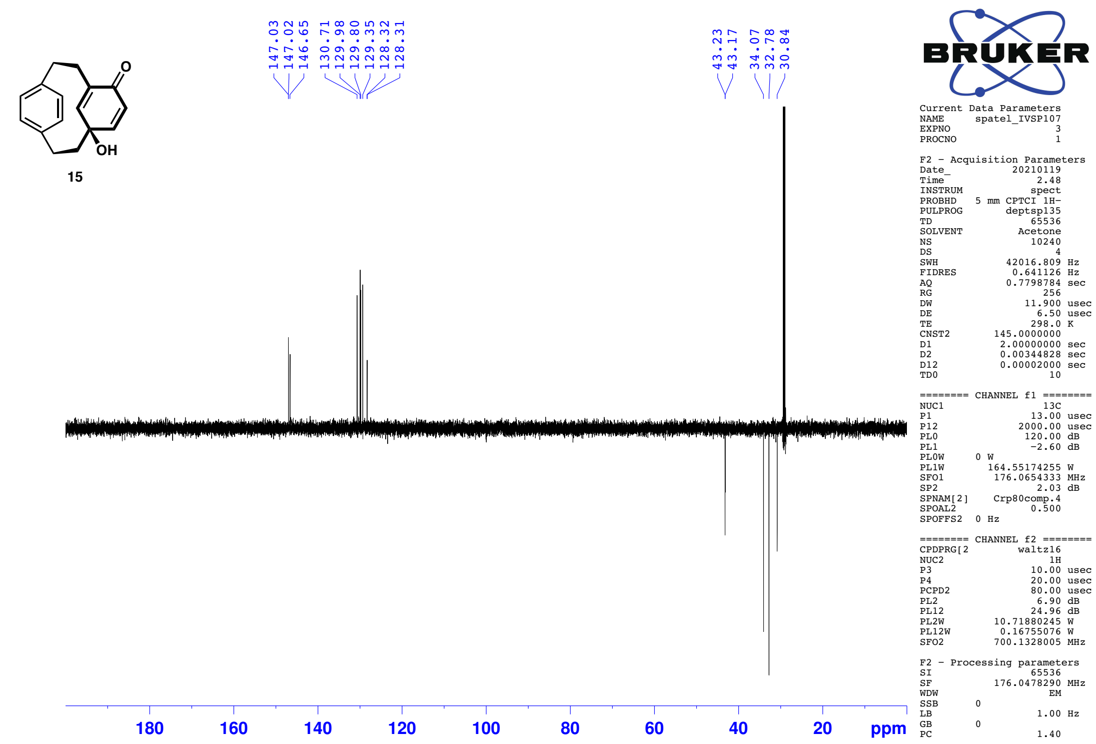

NOESY spectrum of **15** in $(CD_3)_2CO$

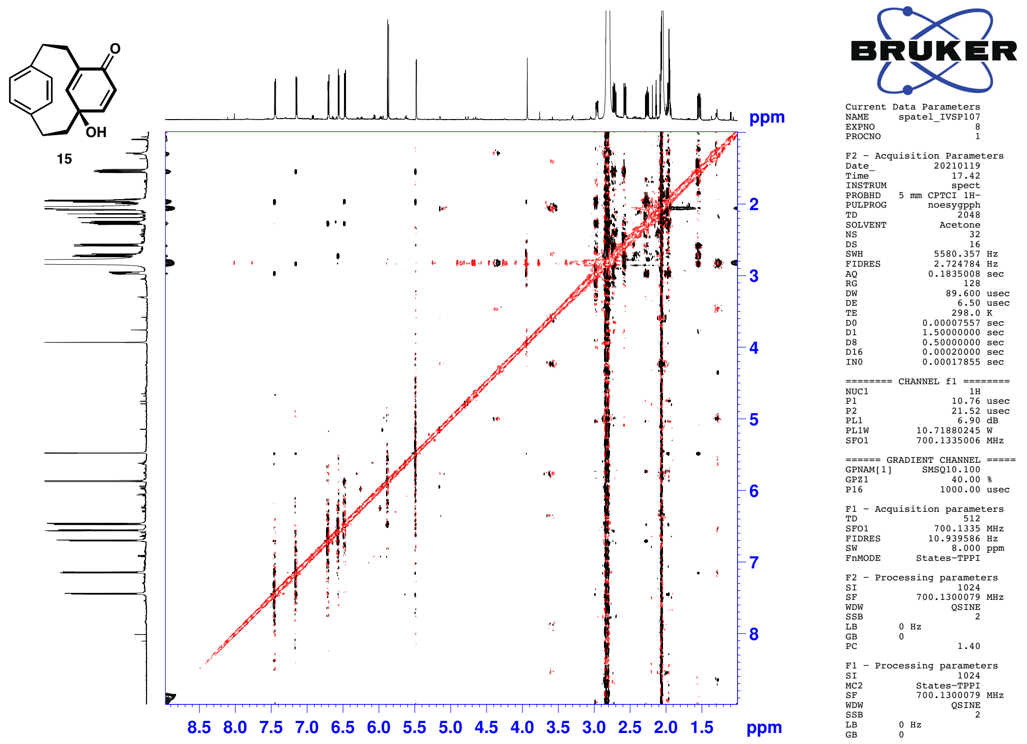

COSY spectrum of **15** in $(CD_3)_2CO$

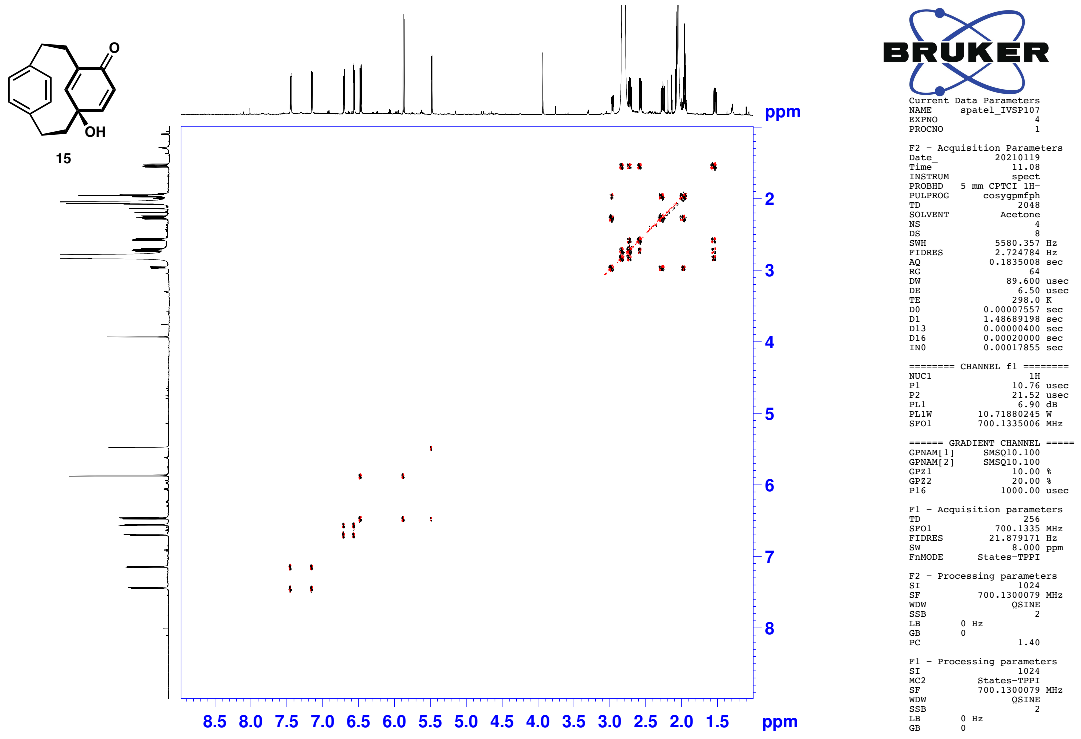

Long range COSY spectrum of **15** in $(CD_3)_2CO$

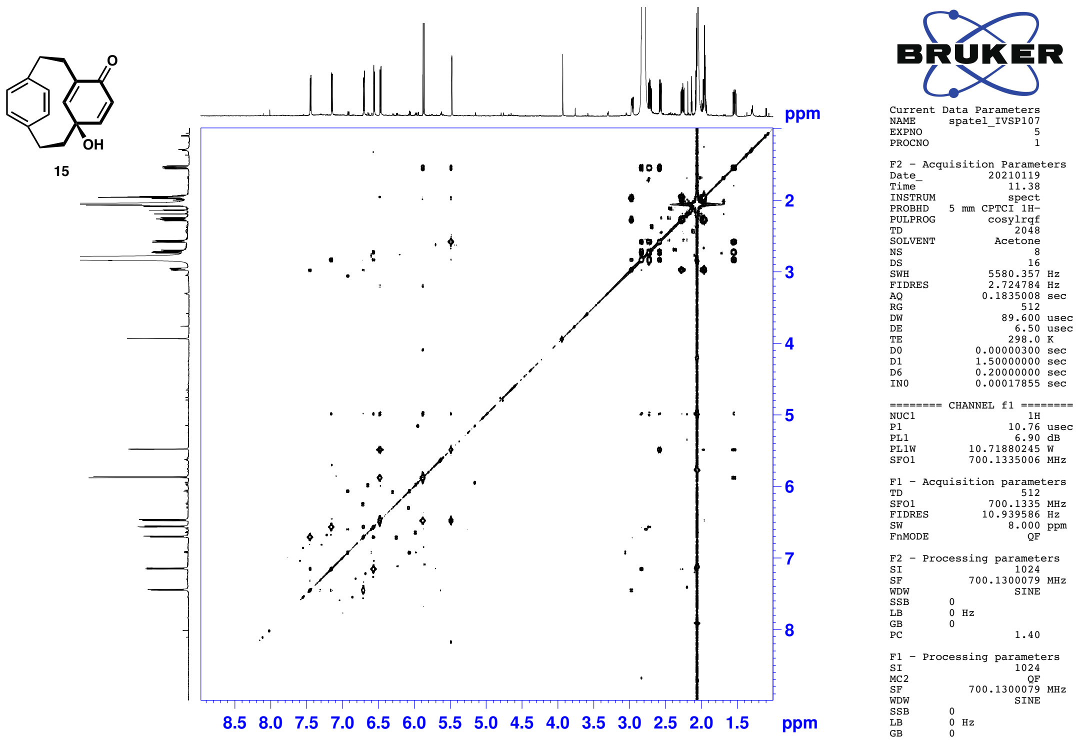

HMQC spectrum of **15** in $(CD_3)_2CO$

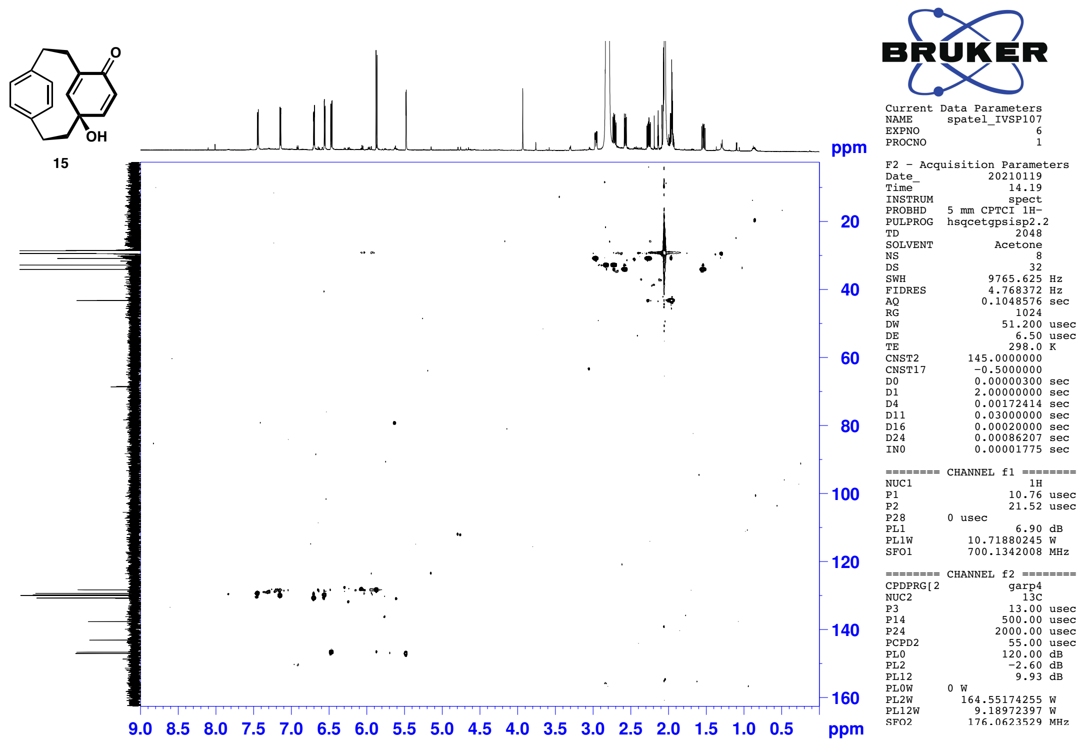

HMBC spectrum of **15** in $(CD_3)_2CO$

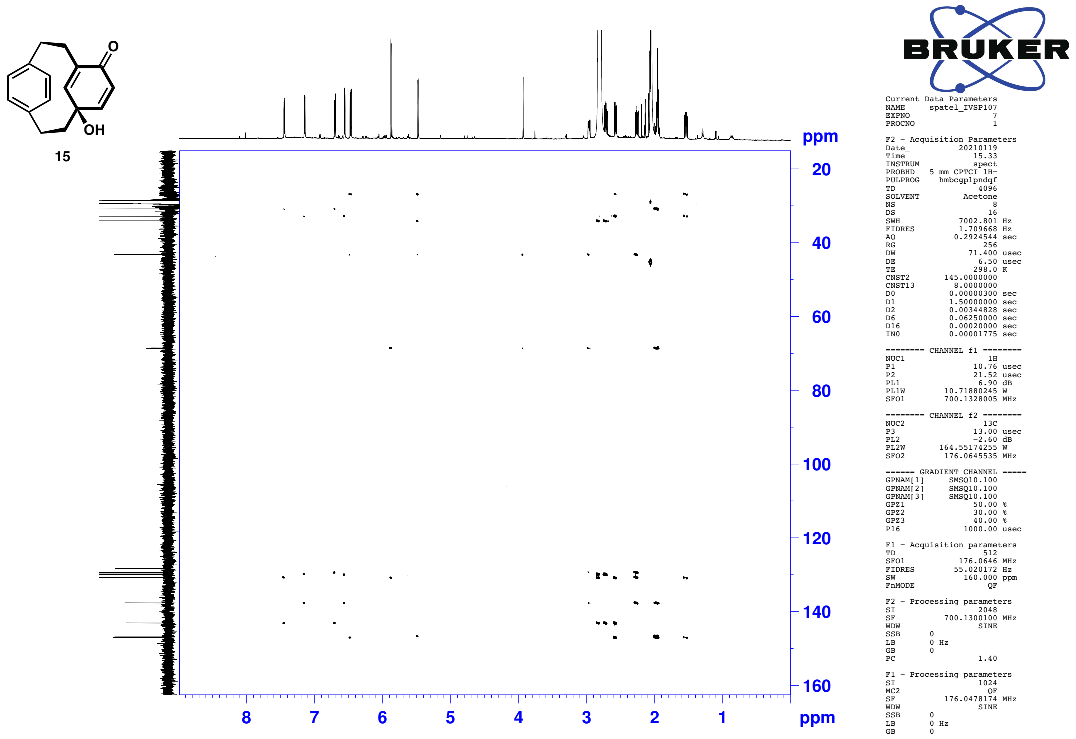

Supplement: File 1 — Metaparacyclophane spectra. [file Beilstein_J_Org_Chem-17-1518-s001.pdf]
